# Demoulding process assessment of elastomers in micro-textured moulds

**DOI:** 10.12688/openreseurope.13716.1

**Published:** 2021-10-06

**Authors:** Elias Liarte, Valentina Zambrano, Leticia A. Gracia, José Ignacio Amor, Marcos Borro, Belén Hernández-Gascón

**Affiliations:** 1 Materials & Components, Instituto Tecnológico de Aragón, ZARAGOZA, 50018, Spain; 2 Advanced Materials, Funditec, Madrid, 28049, Spain

**Keywords:** Demoulding Forces, Rubber, Friction, Adhesion, Reduced Order Model, Sol-Gel Coating, 3D Laser Micro-Texturing, Texture Pattern

## Abstract

**Background:** Micro-texturing is an increasingly used technique that aims at improving the functional behaviour of components during their useful life, and it is applied in different industrial manufacturing processes for different purposes, such as reducing friction on dynamic rubber seals for pneumatic equipment, among others. Micro-texturing is produced on polymer components by transfer from the mould and might critically increase the adhesion and friction between the moulded rubber part with the mould, provoking issues during demoulding, both on the mould itself and on the rubber part. The mould design, the coating release agent applied to the mould surface, and the operational parameters of the moulding/demoulding process, are fundamental aspects to avoid problems and guarantee a correct texture transfer during the demoulding process.

**Methods:** In this work, the lack of knowledge about demoulding processes was addressed with an in-house test rig and a robust experimental procedure to measure demoulding forces (DFs) as well as the final quality of the moulded part, between thermoset polymers and moulds. After the characterization of several Sol-Gel coating formulations (inorganic; hybrid) the influence of several parameters was analysed experimentally, i.e.: Sol-Gel efficiency, texture effects, pattern geometry, roughness and material compound.

**Results:** The results obtained from the experimental studies revealed that texture depth is the most critical geometrical parameter, showing high scatter among the selected compounds. Finally, the experimental results were used to compute a model through reduced order modelling (ROM) technique for the prediction of DFs.

**Conclusions:** The characterization of DFs in a laboratory, with a specific device operated by a universal testing machine (UTM), provided valuable information that allows a fast and optimized introduction of texturing in rubber components. Selection of a novel Sol-Gel coating and the use of the ROM technique contributed to speed up implementation for mass production.

## Plain language summary

The texturing of polymer components, which means to control the outer geometry of the industrial pieces giving them a *"new skin"*, presents an interest as a method to improve their functional behaviour. One outstanding application aligned with the UN Sustainable Development Goals, is to reduce friction and wear, which leads to higher durability and reduction of energy consumption under working conditions. In the EU-funded MouldTex project, this objective is achieved in rubber seals.

For this paper, first, the transfer and texturing during moulding process was analysed. When texturing of rubber components is required at a small scale, well below 1 mm, complex processes are required. Different patterns were initially manufactured into the metallic mould using laser cutting. Second, in order to achieve correct texturing of the rubber component, the demoulding process was studied as well. The influence of different geometry textures, mould coatings and rubber materials were characterized experimentally. Finally, the experimental results were used to compute a mathematical model to facilitate different designs of the textures.

## Introduction

Micro-texturing is a technique applied to different manufacturing processes, with the final goal of improving the functional behaviour of components along their service life ^1– 3^. This approach is used industrially for friction and wear reduction purposes ^4– 6^, in anti-icing applications within the aeronautic field ^7^, in anti-reflective applications within the optical sector ^8^ and for antibacterial treatments in the sanitary field ^9^, among others. The texturing of micro-surfaces can be performed with different techniques, such as lithography, micro-machining, electric discharge ^10^, laser or chemical etching ^11, 12^, depending on the material on which the technique is performed (metal, ceramic, polymer) and the type of texture (micro/nano) ^13– 15^ that needs to be achieved. Among the existing techniques, one of the most promising methods for micro-texturing is laser texturing ^16, 17^, with which the texture is transferred for a large variety of geometries and materials, with both good control and reproducibility ^5^.

There are two main ways to achieve texture on polymers: by applying the texture on previously manufactured components, or by transferring from the mould during the component manufacturing, through either injection moulding ^18– 20^, injection compression moulding, compression moulding ^21^, hot embossing, among others. While the first approach is limited, especially for the production of high-volume components, the second approach is postulated as an encouraging technique for mass production ^3^. In transfer moulding, an adequate texturing on thermosetting polymers is achieved by guaranteeing the correct texture transfer of a negative pattern, so that the desired texture has to be engraved on the mould. Furthermore, in order to facilitate the demoulding of the part, a mould coating is usually required. The operational parameters of the moulding/demoulding process must be adjusted to achieve a replica of the mould texture. The mould design has to further account for dimensional variations of the rubber during the curing and post-curing processes, as well as the coating over-thickness. The demoulding processes of industrial micro-textured components is more complex, because the increase in contact surface between the polymer and the textured mould leads to a higher adhesion, which might cause an incorrect texture transfer. At the same time, non-flat texturing profiles may induce an increment in friction too, so that polymer and mould materials selection is a crucial task for the quality of the texture transfer. Furthermore, requirements on coating-release agents have become more challenging since they have to bear higher forces, caused by the textured surfaces, that affect the renovation procedure. As a further requirement, coating thickness should not distort or hide the textured geometry. Likewise, build-up of deposits on the mould surface, after a usually quite small number of moulding cycles, can be another important issue if demoulding forces (DFs) are too high. Within this background, problems that may arise from demoulding can be complex ^22, 23^; unfortunately, there is no generalized and standardized approach for studying the interaction between the mould surface and the polymer. Therefore, despite the increasing demand for the use of micro-textures, the lack of robust experimental data confirming industrial feasibility, limits the boost of micro-texturing techniques for mass production.

The measurement of DF on industrial moulds is not a common procedure, and precise laboratory test equipment is required. During the moulding/demoulding process, complex phenomena, such as friction and adhesion, are combined at high pressure and temperatures ^24^. Moreover, the presence of textured surfaces, especially in the case of high aspect ratio patterns ^25– 28^, makes the demoulding process even more difficult. It is therefore necessary to carry out experimental tasks to characterize the DF, and to analyze both the coating release agent performance and the material properties’ influence in the demoulding process. Based on authors’ experience with thermosetting materials ^29, 30^, some of the existing experimental methods available in the literature are based on alternatives that are not good enough to characterize the relevant aspects of the demoulding phenomena. One example for coatings is the absence of correlation between the standard test (contact angle of release agent with water or oil) and the DF ^4^. Besides, recent investigations characterized the contact angle of wetting polymers ^23, 28, 31^, but there are still some limitations to evaluate curing polymers along time, under specific pressure/temperature and textured geometries. In addition, the DF must be measured without any contribution of external friction that could modify the results of polymer/coating interaction; the DF coming from different ejector rods, for instance, may contribute to some extra friction or misalignment. An effective solution might be the modification of the compression actuator to extract the piece and measure the DF directly, where the use of a universal testing machine (UTM) is a promising method to completely control the process in a laboratory ^24, 26, 32^.

The study of coating release agents is well documented in the literature. Fluorocarbon or hydrocarbon-based coating materials are the most common anti-stiction coatings ^12, 33, 34^, although they must be properly used to ensure the correct filling of the texture due to their low surface-energy properties. These release agents are usually applied to the mould surface between moulding cycles to enhance release ^35^. Despite their low cost and easy application, standard spray methods present some drawbacks which limit their use to specific operation conditions, and make the development of new release agents to facilitate demoulding in wider operation ranges necessary. Among the emerging release agents, Sol-Gel coatings present promising properties, due to their easy application and potential for achieving very thin layers ( *<* 1 µm) ^36^. In this study, a novel Sol-Gel release agent was developed and applied to the mould surface to facilitate demoulding and to protect the mould from surface contamination.

In order to analyse experimental demoulding data and to predict the system’s outcome under experimentally unexplored working conditions, a reduced order modelling (ROM) technique is used. ROM is a mathematical approach for multivariable problem simplification, and for the obtention of a virtual representation of the real system under investigation ^37– 41^. The selected ROM method was based on the tensor rank decomposition (TRD) technique. TRD is a completely data-driven method for which a problem of N variables can be written as the product of N one-dimensional functions, one for each of the system’s variables, as detailed in a previous publication ^42^. Due to promising results obtained from the application of ROM techniques to both friction reduction prediction and statistical analysis of texture replication ^43^, the method was selected to be applied for DF modelling and prediction, as described in this paper.

**Figure 1.**
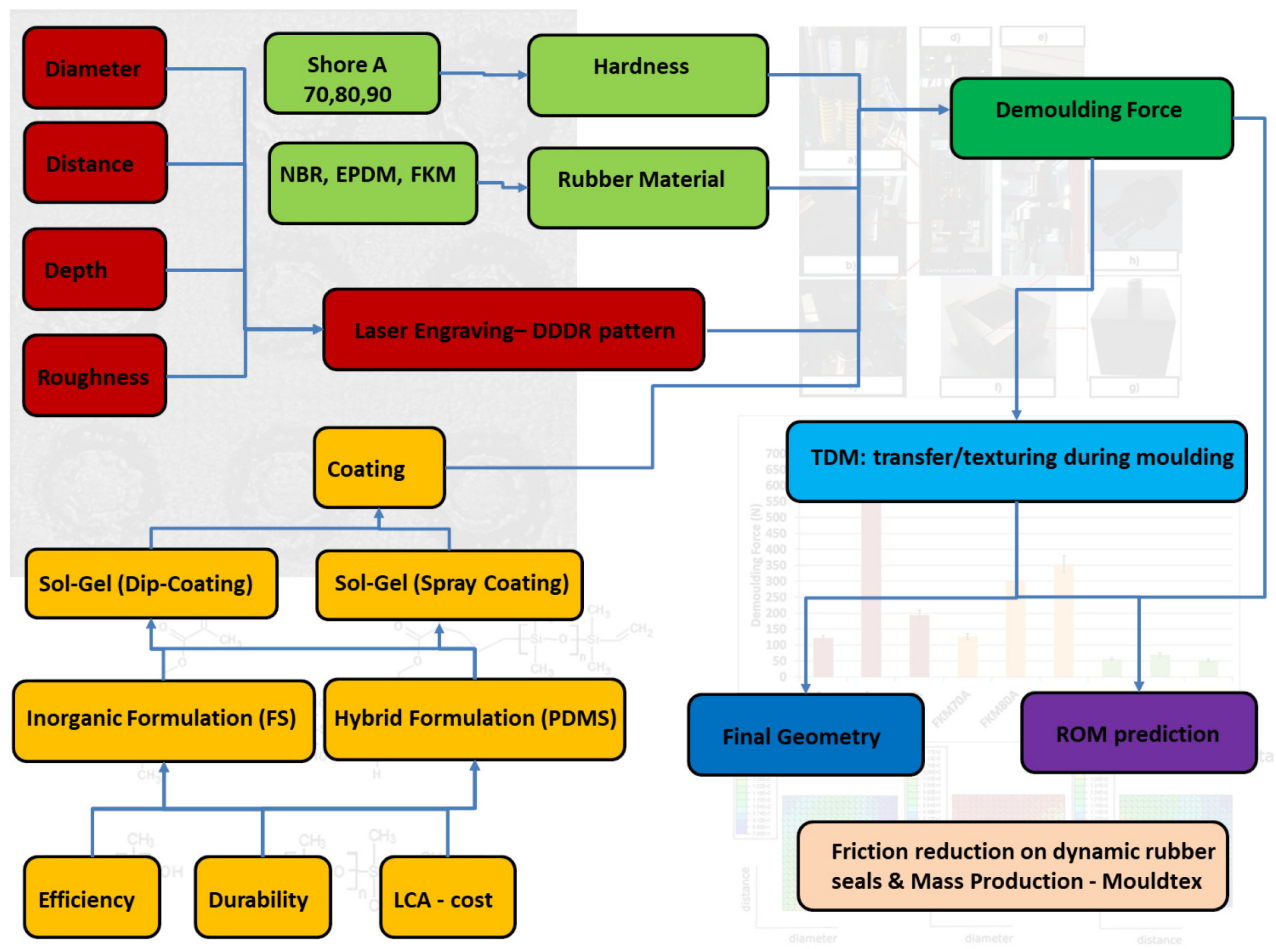
Graphical abstract. Red:texture pattern-related aspects; Green: material compound-related aspects; Yellow: coating-related aspects; Dark green: demoulding-related aspect; Blue: transfer/texturing during moulding-related aspects; Dark Blue: evaluation of the transferred geometry-related aspects; Purple: Reduced order modelling (ROM) prediction of demoulding forces (DFs)-related aspects.

Within this backgroung (see Figure 1. Graphical Abstract), this paper describes a robust experimental procedure to measure DF in a simple cube geometry, between thermoset polymers and coated moulds under similar conditions to those of the industrial manufacturing process. To that end, an in-house test rig, capable of dealing with the deviations that may appear during the manufacturing process itself, was designed and assembled. The coating efficiency was evaluated first to select the most appropriate Sol-Gel and deposition method, which was used for further analysis. Chemical formulations were studied, including inorganic and hybrid formulations with improved durability; in addition, several studies were dedicated to the analysis of the influence of texture, roughness and rubber compounds. In this regard, the micro-texture on the mould was described by a pattern of half-cylindrical micro-protrusions, aka bulges, whose geometry was defined by their diameter, depth and distance between each bulge. These types of texture patterns were selected for the study of friction reduction under dynamic conditions of rubber seals ^4^; DDDR denomination represents the diameter, distance, depth and roughness of the texture, where DDD are hence the diameter, distance and depth. Finally, results from experimental studies were used to create a ROM for real-time DF prediction. The ROM therefore facilitates future designs of textures for industrial applications, based on the force required in the demoulding process.

## Methods

### Samples

***Rubber*.** Nitrile butadiene rubber (NBR), ethylene propylene diene monomer (EPDM) and fluoro carbon based elastomers (FKM) rubber compounds were used to analyse demoulding behaviour. Different hardness levels (ASTM D-2240) were selected for those compounds. Mechanical properties: tensile Strength (ASTM D-412); elongation (ASTM D-412); specific gravity (ASTMD-7920); together with rheological behaviour (ASTM D5289): initial peak (Init) [dNm]; moment lowest (ML) [dNm]; moment highest (MH) [dNm]; time to reach 90% MH (t90) [min.]; were analysed prior to testing (see Table 1). These compounds were selected because of their common use in rubber dynamic seals manufacturing.

**Table 1.** Mechanical properties and rheological behaviour: hardness shore A (HA); tensile strength (TS) [MPa]; elongation (EL) [% ]; specific gravity (SG) [g/cm ^3^]; initial peak (Init) [dNm]; moment lowest (ML) [dNm]; moment highest (MH) [dNm]; time to reach 90% MH (t90) [min.].

Rubber	HA	TS	EL	SG	Init	ML	MH	t90
NBR70	67	20.0	524	1.22	19.83	11.13	38.39	5.15
NBR80	75	14.5	260	1.28	22.57	10.82	39.32	5.37
NBR90	80	17.8	301	1.26	18.62	7.63	38.18	3.59
EPDM70	68	15.0	258	1.13	14.29	5.48	43.90	7.28
EPDM80	77	15.8	261	1.12	20.49	11.34	52.33	7.38
EPDM90	90	23.0	206	1.20	22.09	9.26	55.97	5.59
FKM70	76	12.0	230	1.90	14.98	4.68	32.77	3.72
FKM80	80	7.4	203	1.88	14.77	4.27	28.16	3.58
FKM90	88	14.0	117	1.91	25.59	8.25	70.43	3.91

**Figure 2.**
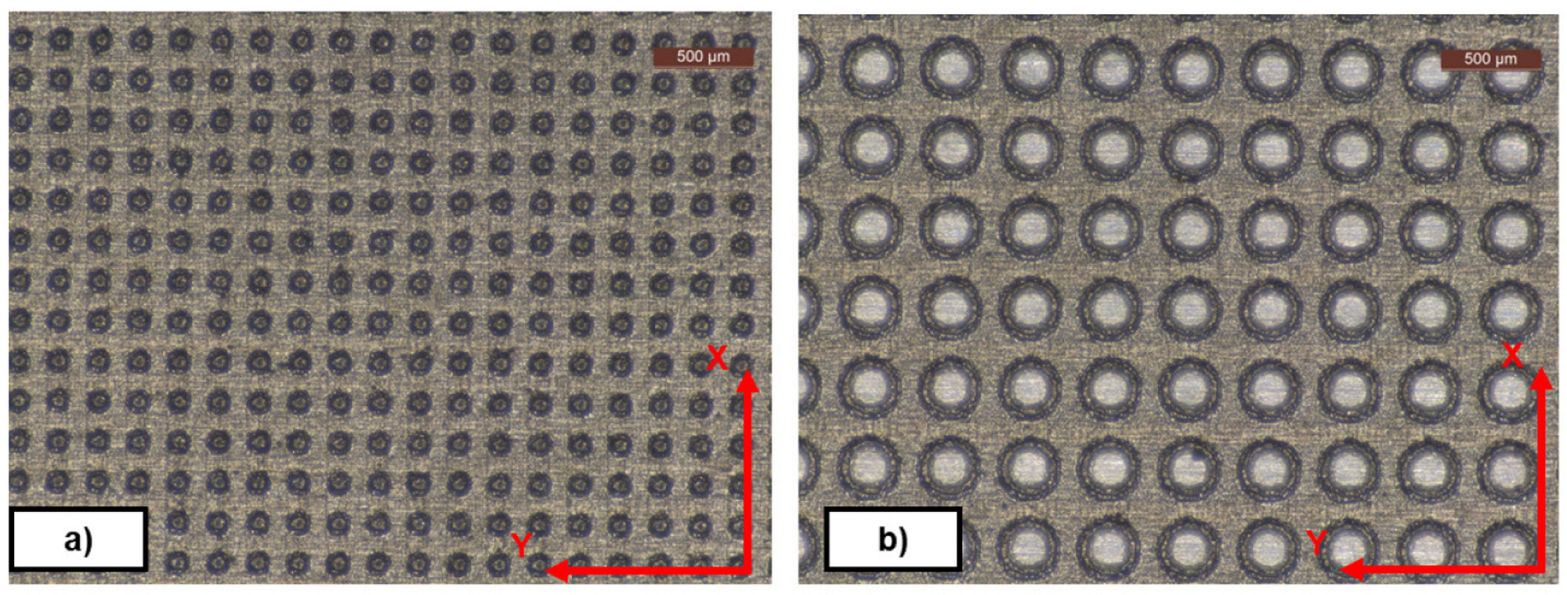
Textured plates. Diameter, distance, depth (DDD) pattern. **a**) DDD 100x100x10 µm; **b**) DDD 300x100x10 µm.

***Metal plates*.** Following strict surface quality requirements, several individual flat 25x25 mm plates made of 1.2312 steel were shop-machined by milling and grinding. Although the set of plates were machined under the same conditions, with R _*a*_=1µm, it should be noted that small differences in the surface roughness of the plates can cause significant differences in the measured DF. In addition, the process to clean and recoat one simple plate is not always straightforward and fast, introducing further sources of errors. Grinding was performed on Y direction (see Figure 2) with an iterative process, in order to obtain the desired roughness. Once machined, the plates were cleaned and coated with oil to prevent them from oxidation. In the demoulding direction X of a lateral plate that shape a cube (see Figure 8f), the demoulding angle is 0 ° degrees, which is the worst situation for the demoulding process with textured surfaces. Nevertheless, if the laboratory demoulding process is achieved with a correct texture transfer, no problems, such as incorrect geometry, build-up of deposits, or fast removal of coating, are expected to occur in the industrial process either.

After the plates were manufactured, they were textured by means of a laser cutter (AgieCharmilles P 1000 U; Pulsed ytterbium fiber laser) to obtain the desired pattern (see Figure 2).

***Coatings*.** Metal plates were coated with a semi-permanent Sol-Gel release agent. Sol-Gel synthesis was based on silica components as main products, which were doped or modified with different components such as titanium, zirconium, fluorine and organosilicons.

Sol-Gel synthesis can be divided into two main methods: inorganic synthesis and inorganic/organic synthesis. The precursors are usually organic compounds, although after a curing treatment most of them result into inorganic polymers. For instance, during the hydrolysis step, tetraethoxysilane (TEOS; used in all formulations) forms inorganic hydroxisilane and, after curing, forms inorganic silica particles or thin films. Other precursors used in some formulations of inorganic Sol-Gel are trimethylethoxysilane (TMES), methyltriethoxysilane (MTES), and fluorosilane (FS). FS is a highly popular compound in demoulding processes to increase the contact angle between polymers and metals, but with a high life cycle ssessment (LCA) impact and high cost ( LCA Software SimaPro 8.2.0.0 with the ecoinvent Life Cycle Inventory (LCI) database).

Fluorinated compounds used as release agents present significant limitations, such as high cost and environmental drawbacks; therefore, hybrid coatings have been postulated as a potential replacement for them. Polydimethylsiloxane (PDMS), an organosilane compound, is extensively used for this purpose. However, the application of PDMS to commercial formulations, with direct application to the mould, shows very short service life, which implies the need for continous reapplication. In spite of good film formation between rubber and mould, it is very easy to remove it, since it is not able to keep its adherence to the mould walls. To solve these limitations, PDMS is added to some hybrid coatings synthesized for improved durability (see Table 5). PDMS is attached to the surface of the steel by means of Sol-Gel synthesis, thus reducing DF and increasing coating service life. Another precursor used for hybrid Sol-Gel formulations is methacryloxypropyltrimethoxysilane (MPTS).

The characterization of the Sol-Gel coatings once they are deposited on the surfaces is performed by different characterization methods: Film Hardness (ASTM D3363), Film Adhesion (ASTM D3359), Water Contact Angle (EasyDrop Contact Angle Measuring Instrument by KRUSS), Film Thickness (profilometer Talystep Taylor-Hobson) and Corrosion Protection (ASTM G102). It’s furthermore necessary to remark the good corrosion protection of the hybrid Sol-Gel coatings; the corrosion rate for the bare substrate (steel) is 0,5358 mm/year while for the MT-P Sol-Gel coating it is 0,00885 mm/year (see Table 2).

**Table 2.** Comparative properties of applied coating on steel surface. Reference (Ref.); Chemical Composition (CC; 1-2- 3 indicates different concentrations); Scratch hardness by Pencil Test (ASTM D3363); Adhesion tests (ASTM D3359); Water Contact Angle (WCA) º; Corrosion rate (mm/year; ASTM G102).

Ref.	CC	D3363	D3359	WCA	G102
FS-3	TEOS/MTES/FS	*>*4H	5B (0%)	63.1	0.304
MT-D	TEOS/MPTS	*>*8H	5B (0%)	62.4	0.034
MT-H	TEOS/PDMS-1	2H-4H	3B (5-15%)	113.0	0.018
MT-J	TEOS/MPTS/PDMS-2	*>*8H	5B (0%)	113.3	0.0002
MT-P	TEOS/PDMS-3	*>*8H	5B (0%)	108.6	0.00885

**Figure 3.**
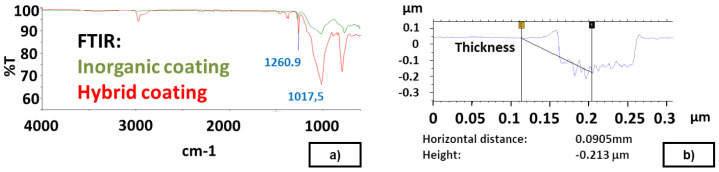
**a**) Sol-Gel FTIR. Inorganic and Hybrid Coating; **b**) Measurement of thickness on a thin Sol-Gel layer.

The chemical characterization of the synthetized Sol-Gel coatings is carried out via common techniques (Fourier-transform infrared spectroscopy (FTIR); Raman spectroscopy; Differential scanning calorimetry (DSC)). Peaks related with the presence of coating on the modified surface were easily identified using FTIR technique. As examples, the following peaks were identified for Sol-Gel coatings: Si-O bond at 1017 cm ^−1^ (anti-symmetric stretching mode), S-O bond at 795 cm ^−1^ (symmetric stretching mode) and Si-C bond (from silanes) at 1260 cm ^−1^ (see Figure 3a). The thickness of Sol-Gel coatings was measured by profilometry on a thin layer on a flat metal plate (Talystep, Taylor-Hobson), resulting in 150 nm and 210 nm for inorganic and hybrid coatings, respectively (see Figure 3b). The application of Sol-Gel by dip-coating modified the initial geometry of the textured moulds since it resulted in a thicker layer (around 2.5 µm in a bulge; see Table 3), probably due to the accumulation of the Sol-Gel coating on textured plates, or on the "walls" of the protrusion.

**Table 3.** Estimation of Sol-Gel thickness by comparison of "Uncoated vs Coated" textured plate. Dip-coating application. Diameter, distance, depth (DDD) pattern. Ref-1: DDD 100x100x15 µm. Ref-2: DDD 200x200x15 microns. Measures in µm. Mean value and standar deviation (SD).

Reference	Uncoated		Uncoated		Coated		Coated	
	Diameter	SD	Distance	SD	Distance	SD	Distance	SD
Ref-1	132	7.1	72.5	2.1	138.5	4.9	67	8.5
Ref-2	238	5.7	175.5	3.5	242	4.7	171.5	9.2

### Experimental methods: preliminary and complementary analysis

***Texture and pattern transfer*.** Evaluations of the geometry transfer from the mould were performed on NBR70A rubber cubes with a Leica DMS1000 (2D) digital microscope, an Inverted Zeiss LSM 880 laser scanning confocal microscope (3D) and a Form Talysurf 120i Taylor-Hobson Roughness Tester / Profilometer (see Table 4). The selected objective for the transfer quality was to reach a steady transfer with differences below 10% of the diameter and distance nomimal values ^4^. The results showed minor differences: below 10% from the nominal value of diameter and distance in all cases (around 5 µm). Similar absolute value results were found for depth transference (around 5 µm; Depth in the range of 10 – 20 µm), although in this case, the deviations were higher in percentage. Later adjustments were carried out in laser texturing to set the offset for mass production. Transfer evaluation showed similar trends for FKM90A rubber.

**Table 4.** NBR70A 2D transfer evaluation. Measures in µm. TEDiam: transfer evaluation of diameter; TEDist: transfer evaluation of distance.

plate	Diameter	Distance	Diameter	Distance	TEDiam %	TEDist %
	metal a)	metal b)	Rubber c)	Rubber d)	(a-c)/a	(b-d)/b
1	144	107	148	98	2.92	- 9.10
2	204	100	188	106	-7.67	6.38
3	145	157	146	145	0.62	-7.44
4	204	152	199	143	-2.22	-6.04
5	172	83	168	77	-2.46	-6.20
6	221	86	211	84	-4.35	-1.94
7	170	134	169	125	-0.55	-6.46
8	222	132	216	128	-2.59	-3.25
9	184	117	182	114	-1.09	-2.65
10	185	117	178	118	-3.37	0.27
11	184	116	182	114	-1.09	-2.50

**Table 5.** Preliminary test. Durability of the coating. FKM90A.

Coating	Chemical Composition	Service Life (Cycles)
MT-D	TEOS/MPTS	10
MT-H	TEOS/PDMS-1	5
MT-J	TEOS/MPTS/PDMS-2	8
MT-P	TEOS/PDMS-3	40
FS-3	TEOS/FS	10
Commercial	Rocol® PR Spray	3
Bare Steel	Bare	1

**Figure 4.**
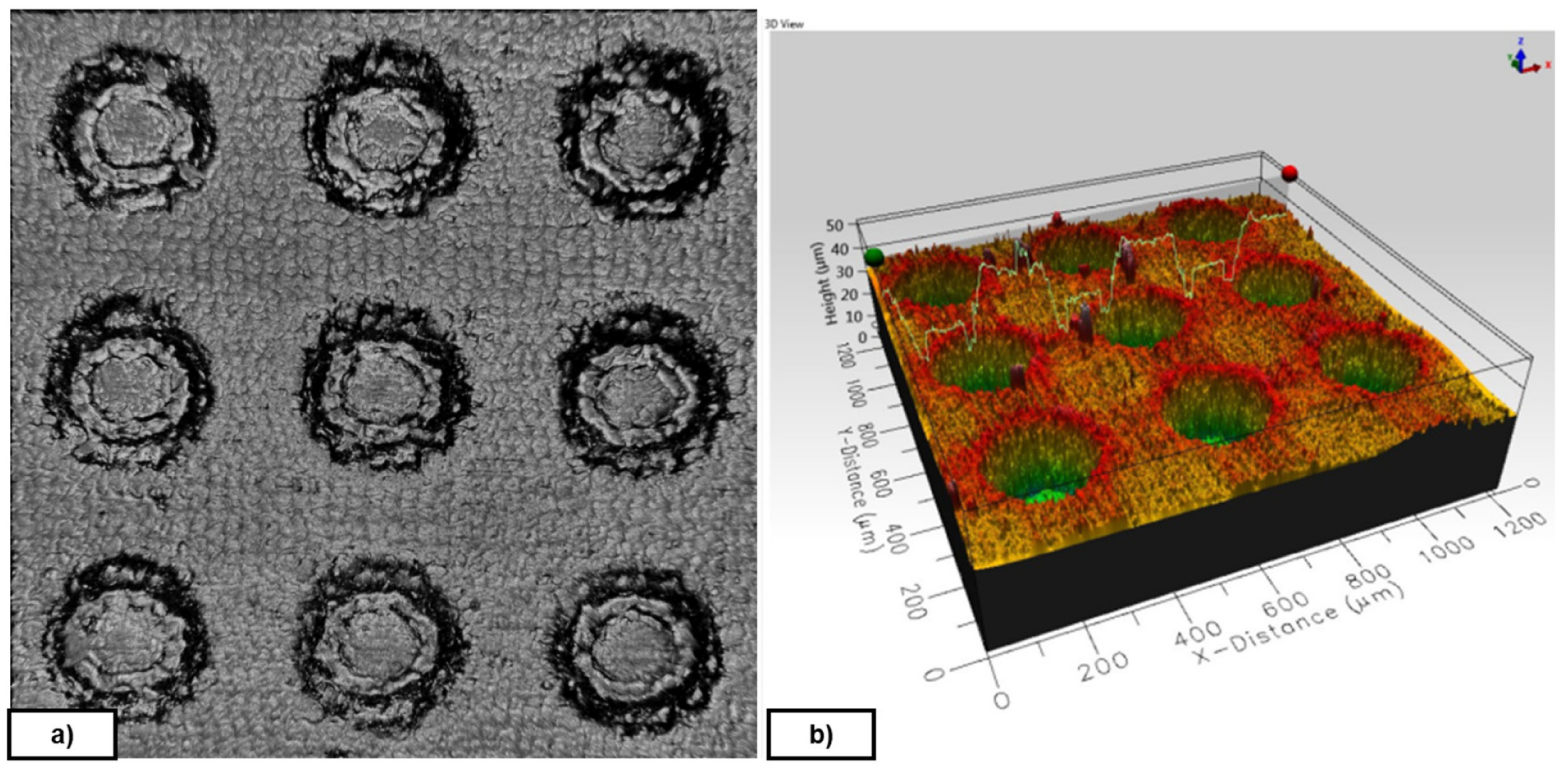
Diameter, Distance, Depth (DDD) pattern. **a**) Textured NBR70A rubber (DDD 175x125x10 µm); **b**) FKM90A rubber (DDD 300x100x10 µm) - picture by Optimal-Optik.

An example of rubber cube surface and different textures observed after the demoulding process is depicted in Figure 4.

**Figure 5.**
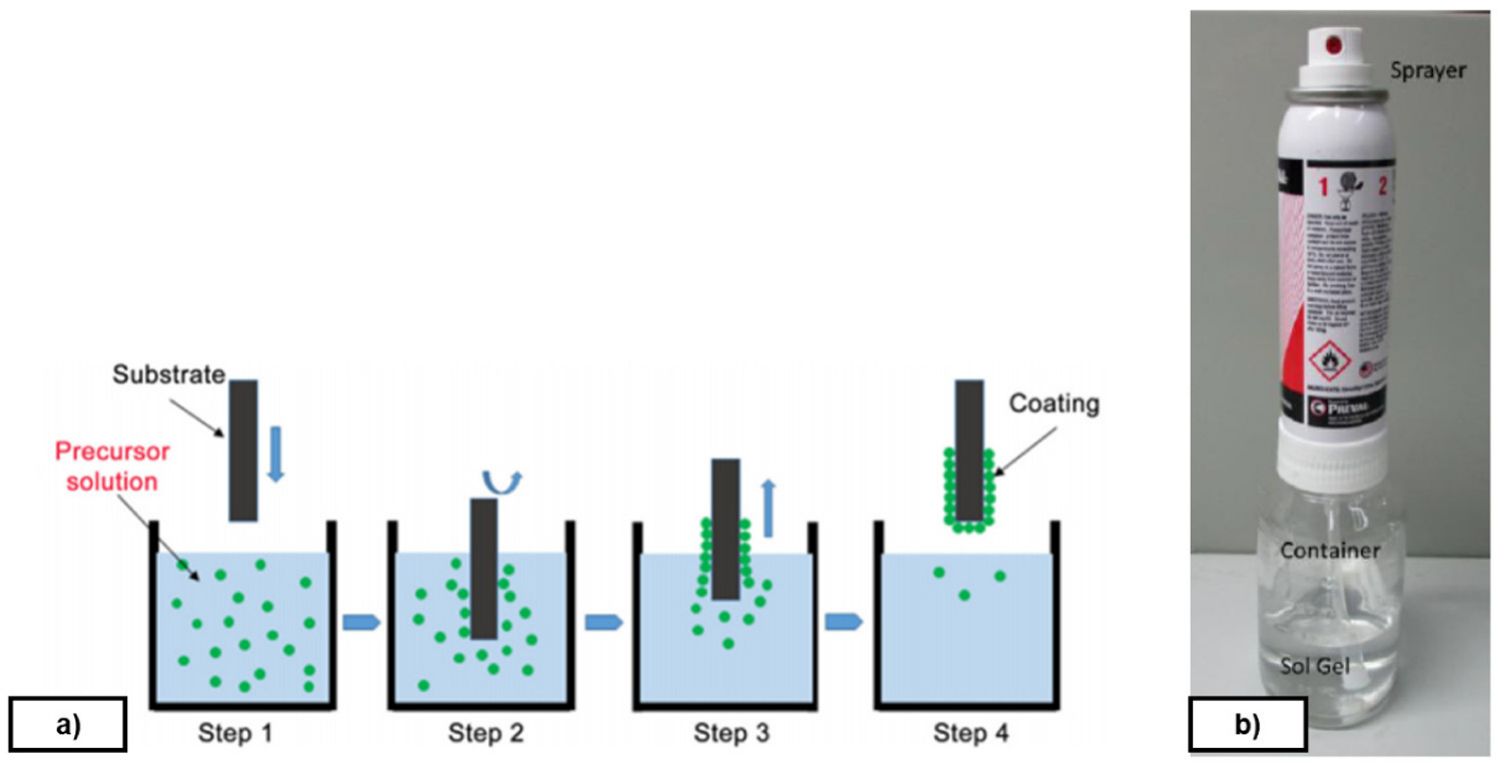
**a**) Sol-Gel application procedure by dip-coating; **b**) Refillable spray system.

***Sol-Gel deposition method*.** Sol-Gel can be applied by several methods. In this work, both spray and dip-coating techniques are studied. Dip-coating is the preferred method according to experimental results reported in Figure 11; it is a simple, low-cost, reliable and reproducible method. Dip-coating consists on the deposition of a wet liquid film by substrate immersion into a hydrolysable metal compound solution and its extraction at constant speed (see Figure 5a). A homogeneous liquid film is formed on the substrate and, after drying at room temperature, the formed film is hardened at 180 °C. The application of a semi-permanent mould release agent type protects the metal from oxidation, reduces the coefficient of friction with the rubber and reduces the adhesion of the polymer to the mould during the curing process.

Spray method is used with a refillable system which allows the user to mix up any kind of Sol-Gel and turn it in to sprayable material through venturi vacuum process (see Figure 5b). It is important to highlight that this system contains propane and butane which are flammable gasses. Droplets obtained with this system are bigger than dip-coating, and therefore resulting coatings are thicker. It is nevertheless possible to obtain continuous coatings showing good performance. Besides, spray method is cheap because it does not need investment for industrial uses and lots of coatings can be deposited.

**Figure 6.**
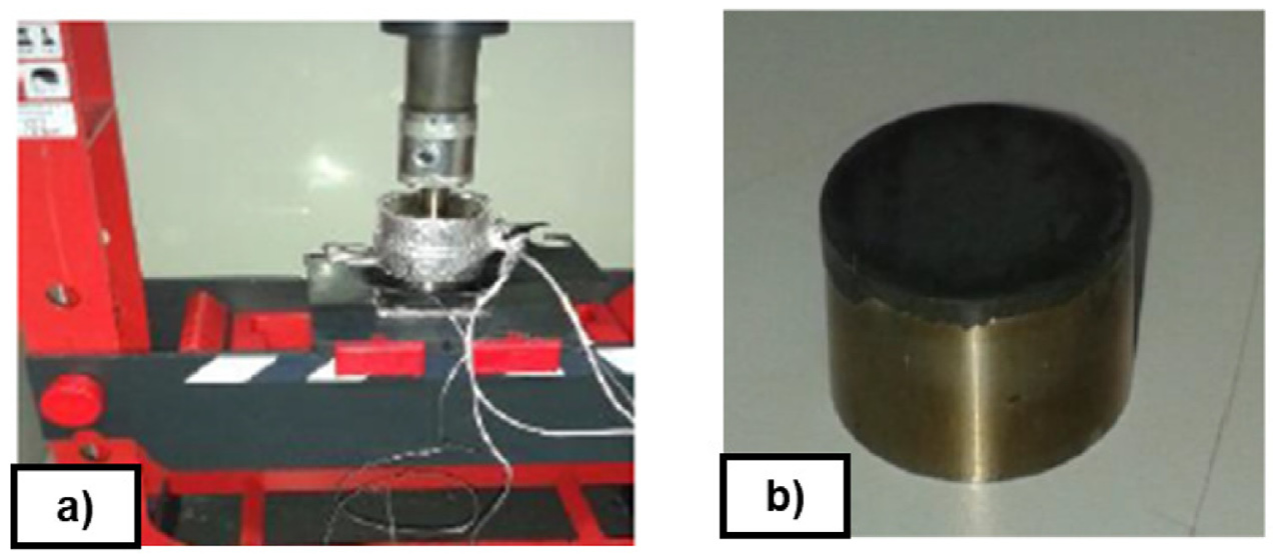
**a**) Hydraulic press; **b**) Disc of rubber.

***Durability estimation of Sol-Gel*.** One of the main limitations of the coatings used for demoulding processes is their durability. Fast removal of the coating requires costly renovation procedures in the mould. Therefore, the durability improvement of the semi-permanent Sol-Gel coatings becomes a key factor. To evaluate the durability of different Sol-Gel formulations with different precursors and concentrations (see Table 2), in a qualitative way, some preliminary tests were carried out at laboratory scale. Around 700 mg of uncured rubber FKM90 were heated and pressed between two cylindrical stainless steel plates at 170°C and 2900 bar, in a laboratory press for 15 minutes (see Figure 6). These non-textured discs were previously coated with selected release coating by dip-coating technique. Results were evaluated in terms of demould or no demould without visual defects.

**Figure 7.**
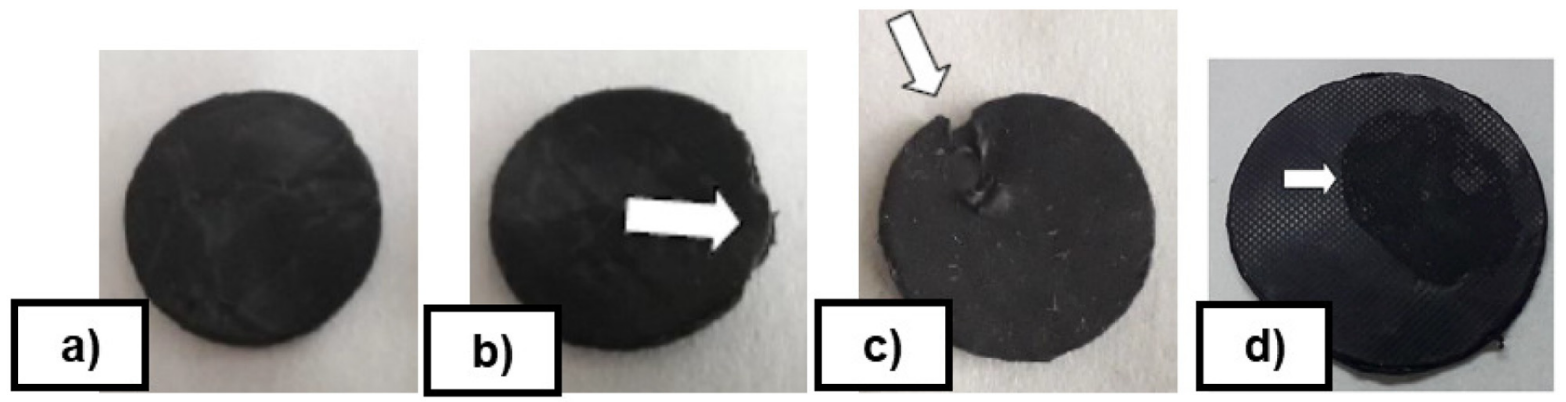
**a**) Disc without defects; **b**) High deformation to demould the disc; **c**) Build-up material on the dics; **d**) Superficial defect.

A comparison of the release performance of the synthesized coatings with different precursors and concentrations was done (see Table 5). The comparison was carried out by testing the number of vulcanizing cycles without sticking the rubber (FKM90A) on a non textured disc covered with each Sol-Gel formulation. The commercial coating release agent Rocol ^®^ PR Spray and bare steel tests were added as references. The resulting service life is the number of cycles achieved until reaching a failure or visual defect in the demoulding of the rubber disc from the coated metal disc (see examples of failures in Figure 7b,c,d). These durability tests were used for screening the coatings.

The inorganic coating FS-3 withstood up to 10 vulcanization cycles, whereas the hybrid coating MT-P improved on this result to up to 40 cycles in total. MT-P formulation, in terms of service life (cycles) showed the best durability performance, and best corrosion protection. Due to these results, MT-P coating was selected for final industrial application ^4^. Durability results were in agreement with the results presented in Figure 14.

### Demoulding forces measurements

**Figure 8.**
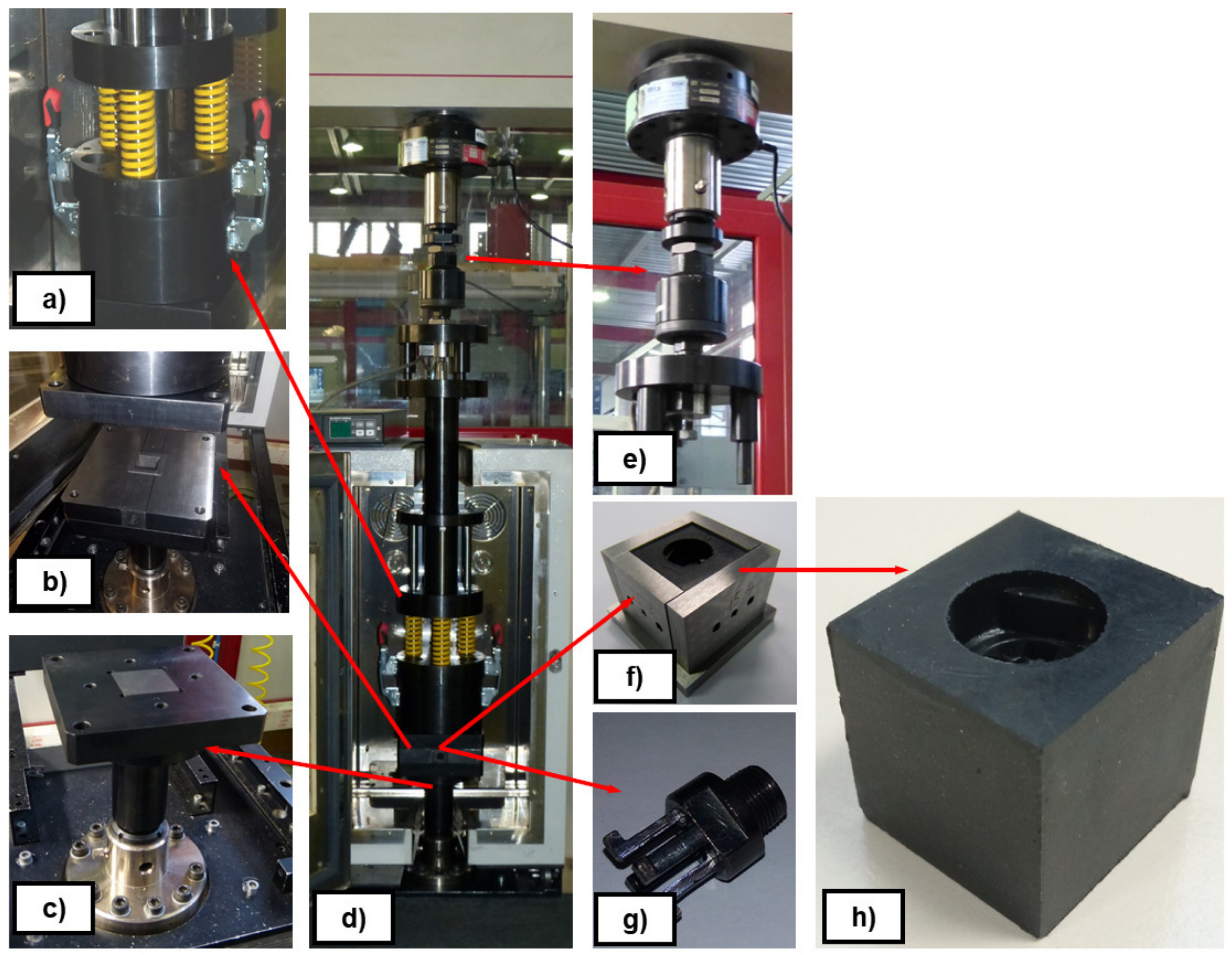
Scheme of the demoulding test rig assembly. **a**) transference chamber; **b**) mould cavity; **c**) lower mould wall; **d**) general assembly; **e**) measurement system on UTM crossbeam; **f**) plates and sample configuration; **g**) metallic undercut; **h**) textured rubber cube.

**Figure 9.**
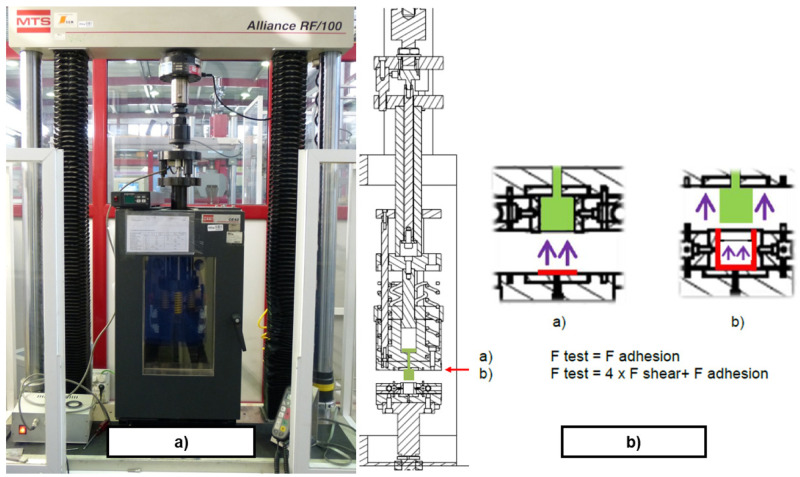
**a**) General view of the demoulding test rig; **b**) Schematic sectional view of the sample area with main frictional forces involved.

**Figure 10.**
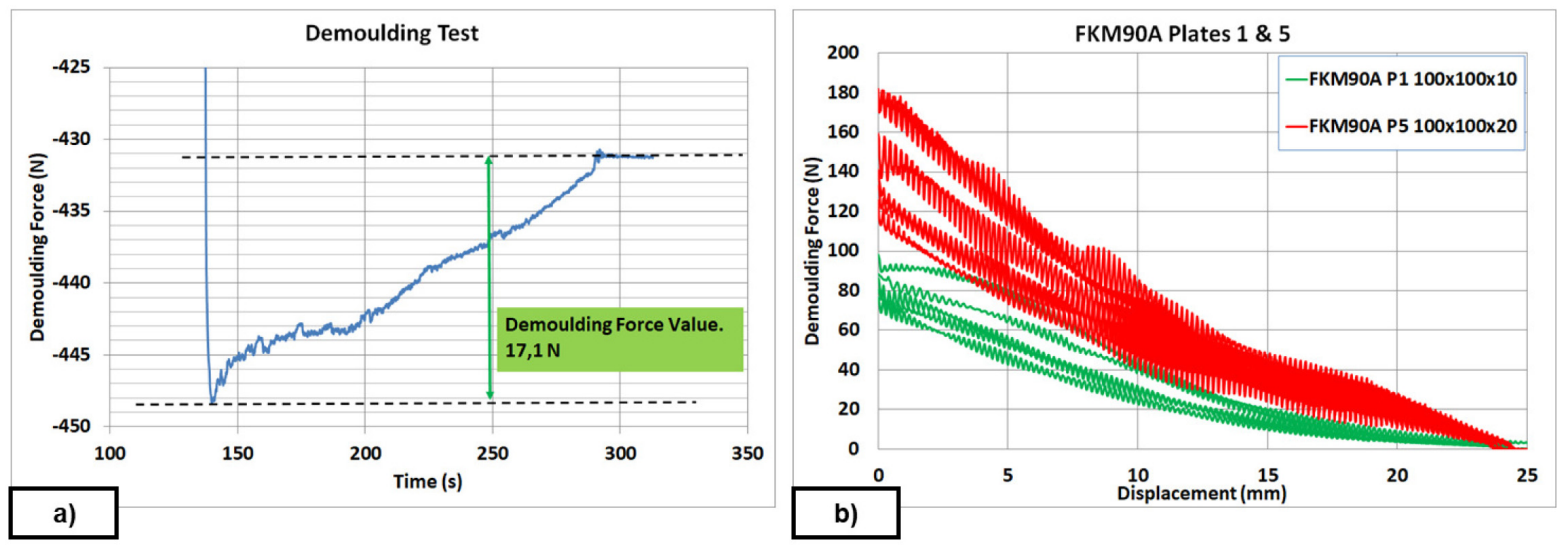
**a**) Demoulding force extraction curves in a flat plate; **b**) Post-processed force extraction curves for FKM90A in a textured plate.

***Demoulding test rig*.** The measurement of DF on thermosetting materials was performed experimentally by replicating the industrial process of transfer moulding through a small and simple cube-shaped mould, the internal walls of which were either non-textured or textured, depending on the study case. The designed test rig intended to replicate a transference mould with the capability of measuring DF. In order to simplify the test rig design, the implementation of specific test fixtures were designed and manufactured to be assembled in a UTM (MTS Alliance RF/100). That way, force, displacement and temperature could be controlled via the UTM software. The designed test fixture, see Figure 8, was assembled with the UTM and the climatic chamber (MTS CE42), as shown in Figure 9a. Several thermocouples control the temperature of the system to ensure the steady behaviour of the metallic parts. A MTS 100 kN load cell was used to transfer the rubber to the mould and a HBM U2B load cell of 2 kN was used to measure DF. All signals (force, displacement and temperature) were integrated in real-time with an HBM Quantum amplifier. An example of a demoulding curve is depicted in Figure 10. The configuration of the demoulding test rig allows the user to measure all parameters continuously during moulding (forces *>* 20 kN) and demoulding (force range: 10 – 1000 N) processes without any additional friction force. The assembly of the plates allowed manufacturing the rubber cube, which represents the selected reference geometry for comparing the texture transference from the metal cube to the elastomeric component (see Figure 8f).

***Cube-sample manufacturing and demoulding tests*.** The first step to be performed for the demoulding test, was the preparation of the raw material. The uncured compound was prepared to be fed into the transference chamber in order to obtain square portions of about 25 – 30 mm size, up to 40 g weigth. The selected size allowed homogenous filling of the transference chamber by means of a feeding pipe, which helped to reach the transference chamber through the free space between the springs used for closing the mould cavity. Following material supplier’s recommendations, NBR, FKM and EPDM uncured compounds were transferred to the mould at 170 °C. The velocity of material transference was an additional parameter to take into account. The higher the material transference velocity, the higher the avoidance of material pre-vulcanization (1 min maximun), although the probability to generate high load peaks increases when the injection piston touches the raw material, due to a low temperature in the latter. After several trials, the transference of the material was performed at 60 mm/min. The compression force for the introduction of the material into the mould reached values up to 50 kN, depending on the raw material. Finally, the force was fixed to 25 kN and the sample was cured at 170 °C for 10 minutes.

Demoulding tests were carried out as follows: for each specific study, up to seven repetitions were performed with the designed fixture assembled at the UTM, with very precise measures to evaluate the DF. Once the sample was cured and ready for demoulding, the demoulding test started at 170 °C with a 10 mm/min rate, being force/displacement continuously measured with a 2 kN load cell (see Figure 10a) and a Linear Variable Displacement Transducer (LVDT). The DF was computed as the difference between the highest force peak value and a later stabilized force value, which corresponds to the added weights of the tools and the rubber sample. When textured plates were analysed, oscillations were noticed in the curves due to the friction between the rubber and the metal protrusions (see Figure 10b). The first values obtained from each performed test are not included in the analysis and tables because they were considered as outliers. 

***Demoulding test: analysis of Sol-Gel formulation and efficiency*.** In a mass production process, the coating release agent facilitates the correct transfer of the geometry/texture to the final manufactured components; therefore, lower chemical adhesion and coefficient of friction reduction are expected. In most cases, industrial manufacturers apply the release agent only when demoulding issues appear. Unfortunately, there is no standard approach for DF reduction. The process is not often optimized due to the limitations of the release agent and renovation procedure. Nevertheless, it is still possible to reduce costs in the application of a coating by optimizing the chemical formulation and selecting the ones with higher durability and efficiency. Minimization or removal of the fluorinated derivates is an interesting option in terms of cost reduction, and fosters a clear improvement in LCA. In this paper, the reduction in mould release forces, thanks to the use of novel Sol-Gel coatings, could reach up to 50%, depending on roughness, textures and the type of rubber compound used.

The efficiency of the coating release agents was evaluated in a laboratory through demoulding tests with the in-house demoulding test rig, described in Figure 8, by comparing force values measured for both non-coated and coated moulds. A FTIR analysis of the coating chemical components was performed to check durability, before and after test completion, showing that test repetition does not cause fast degradation of the coating.

To analyze the influence of the inorganic coatings on the DF, a design of experiments (DoE) was executed with different formulations of the chemical part of the coating to minimize the presence of fluorinated components (see Table 6). Likewise, coated and non-coated flat mould plates were tested trough five repetitions to prove their reproducibility. The material used in the experiments was NBR70A rubber. The DoE was planned with a statistical software (Minitab16 ^44^); a 2-level factorial design (1/2 fraction) including 3 factors and 1 center point was selected (-1: low concentration; 1: high concentration; 0: intermediate concentration; values of concentrations were under confidential restrictions by Funditec). The precursors tested were TMES, TEOS, FS and MTES, TEOS, FS.

**Table 6.** Design of experiments (DoE) for chemical formulations of inorganic Sol-Gel coating.

Reference	Plate	TMES	MTES	TEOS	FS
FS-1	1	-1		-1	-1
FS-2	2	1		1	-1
FS-3	3		-1	-1	-1
FS-4	4		1	1	-1
FS-5	5	-1		-1	1
FS-6	6	1		1	1
FS-7	7		-1	-1	1
FS-8	8		1	1	1
FS-9	9	0		0	0
FS-10	10		0	0	0

The efficiency of the coatings was evaluated according to Equation 1.


$$\mathrm{Efficiency}\text{=}100\frac{DF_{u}-DF_{c}}{DF_{u}}\%\;(1)$$

where, *DF _c_* and *DF _u_* are the demoulding forces for the coated and non-coated plate, respectively.

***Demoulding test: texture pattern analysis*.** Following the results obtained from preliminary tests, NBR70A rubber, the most easily demouldable compound, was selected to analyse the influence of the texture patterns on the DF. A DoE was carried out with different combinations of the protrusions (DDD), while the roughness (R) of the metal plates was fixed to R _*a*_ = 1 µm. Results were obtained using the same batch of NBR70A rubber in order to avoid further uncertainties. The DoE was planned (see Table 7) with a statistical software ^44^; a 2-level full factorial design including 3 factors and 1 center point with 3 replicates was selected. Values of DDD were selected according to finite element analysis (FEA) simulations ^43^, where friction reduction of textured rubber seals in dynamic conditions was expected and finally achieved. We found that the texturing of the metal plates increased the DF, which could compromise the correct texture transfer. Moreover, according to the results obtained from preliminary tests, FKM90A rubber, the most arduously demouldable compound was selected under the same considerations. Again, the variables’ range was defined through new FEA simulations where friction reduction of textured rubber seals in dynamic conditions were expected and finally achieved.

**Table 7.** Diameter, distance, depth (DDD) combinations for NBR70A and FKM90A rubbers (values in µm). Reference (Ref.).

Plate	NBR70A	NBR70A	NBR70A	Plate	FKM90A	FKM90A	FKM90A
Ref.	Diameter	Distance	Depth	Ref.	Diameter	Distance	Depth
P1	125	125	10	P1	100	100	10
P2	175	125	10	P2	300	100	10
P3	125	175	10	P3	100	300	10
P4	175	175	10	P4	300	300	10
P5	125	125	20	P5	100	100	20
P6	175	125	20	P6	300	100	20
P7	125	175	20	P7	100	300	20
P8	175	175	20	P8	300	300	20
P9	150	150	15	P9	200	200	15
P10	150	150	15	P10	200	200	15
P11	150	150	15	P11	200	200	15

***Demoulding test: roughness modification by laser*.** In order to analyse the influence of roughness in the demoulding process and its modification by laser ablation, five sets of new metal plates were manufactured by milling and grinding in a flat configuration. Four of the metal plates sets were laser-treated to provide specific surface roughnesses (see Table 8), while the remaining set, with no laser surface modification was used as reference. The values of roughness were selected according to applications where either low or high friction was required.

**Table 8.** R *_a_* roughness selection for evaluating its influence on different plates (values expressed in µm). Milling and grinding (*); Laser (**).

Plate 1 *	Plate 2 **	Plate 3 **	Plate 4 **	Plate 5 **
0.4	0.5	1	2	5

Machining by milling and grinding showed some variability on the demoulding force for uncoated flat plates (see Table 10). Hence, an interesting approach when high precision control is required, is the use of laser technology to control roughness as an alternative to machining or sandblasting.

All plates were measured with a profilometer and were coated with MT-P Sol-Gel before starting the demoulding test. The features of the different sets of non-textured metal plates were recorded in Table 9. The main parameters describing the roughness were line parameters R _*a*_ and R _*z*_. It should be noted that the ratio R _*z*_ /R _*a*_ appeared similar for all machining processes, which suggests that laser processes do not worsen the final quality of the surface roughness.

**Table 9.** Roughness evaluation for different plates (in µm).

Plate	Manufacture	Coated	Direction	R _*a*_	R _*z*_	R _*z*_/R _*a*_
1	Milling & Grinding	N	Long	0.15	1.03	6.87
1	Milling & Grinding	N	Long	0.27	2.64	9.78
1	Milling & Grinding	Y	Long	0.16	1.27	7.94
1	Milling & Grinding	Y	Cross	0.13	0.91	7.00
2	Laser	N	Long	0.41	2.75	6.71
2	Laser	N	Cross	0.38	2.70	7.11
3	Laser	N	Long	1.24	7.52	6.06
3	Laser	N	Cross	1.20	7.25	6.04
4	Laser	N	Long	2.50	16.80	6.72
4	Laser	N	Cross	2.54	16.46	6.48
5	Laser	N	Long	5.88	35.46	6.03
5	Laser	N	Cross	5.76	33.66	5.84

**Table 10.** Sol-Gel efficiency; flat mould. Water contact angle (WCA); demoulding force (DF); coefficient of variation (CV).

Plate	WCA [°]	DF [N]	CV [%]	DF [N]	CV [%]	Efficiency [%]
	Coated	Uncoated		Coated		
**1**	74.4	23.5	25	13.9	12	40.6
2	105	21.1	17	21.1	6	-0.3
3	63.1	28.2	8	13.3	15	52.7
4	95.8	16.4	6	15.8	10	4.1
5	95.3	15.0	6	12.7	8	15.0
6	80.4	25.3	15	19.4	5	23.5
7	103	24.4	34	22.8	8	6.5
8	101	23.0	14	20.2	9	12.3
9	103	30.3	2	17.8	11	41.4
10	103	22.7	9	15.4	7	32.3

***Reduced order modelling (ROM)*.** The use of ROM is an interesting approach to achieve a real-time estimation of the DF and to predict these values under experimentally unexplored conditions. The data resulting from the experiments described in Figure 12 and Figure 13, and reported in the Data availability section, were further analysed to compute a ROM model for the DF, for each studied material. The ROM used for this analysis was based on the tensor rank decomposition (TRD) technique, where the system parameters’ effect on the output are decoupled in such a way that it is possible to write the system’s response in the form of a series expansion; see Equation 2.


$$F(v_{1},...,v_{N})=\sum_{m=1}^{M}\alpha_{m}\;\prod_{n=1}^{N}f_{m,n}(v_{n})\;(2)$$

where *M* is the order of approximation of the ROM model and *α _m_*, *m* = 1, . . . , *M* are weighting coefficients. The first term in the expansion sum shown in Equation 2 represents a first approximation of the system, while the following terms are corrections to the previous terms and will generally have lower coefficients. The software library Twinkle was used to perform ROM, and is available on GitHub ^45^. Twinkle has been previously described in 42.

## Experimental results

### Evaluation of Sol-Gel as a release agent

Efficiency assessment of the inorganic Sol-Gel coating for NBR70A material results, according to the DoE in Table 6, are reported in Table 10. DF depends on different factors, which may cause outstanding variations in the results. In order to minimize these variations, one single NBR70A material batch was used for these tests. As already mentioned, the temperature required to process uncured batches was 170 °C for 10 min; the cured state was achieved before the sample was extracted. Ten sets of flat plates were machined under the same conditions, with R *_a_* = 1 µm.

In the DoE presented in Table 6, ten different formulations were tested, each one on a single set, to assess the efficiency of the different coatings under study (precursors TMES, MTES, TEOS and FS). Uncoated flat plates showed variability (coefficient of variation (CV) = 30%) in the DF due to compound variations and to the machining process (milling and grinding), even when R *_a_* was set to 1 µm as for the other analysed plates. According to the test results, chemical formulation of plate 3 (FS-3) reduced the DF by up to 50% in a non-textured mould (flat plates). In addition, four formulations reached a DF reduction higher than 30% (FS-1; FS-3; FS-9; FS-10). This allows a compromise between friction reduction and lower quantity of fluorinated derivates, with consequent improvement of LCA evaluations and cost reductions. Most of the formulations (excluding FS-2) generated a positive effect on the DF reduction. These results do not reveal a correlation between efficiency and water contact angle test on the coated plates (FS-7). Formulations with lower concentrations of FS showed high efficiency (FS-1; FS-3), which suggests a possibility to replace these chemical component, and hence reduce cost and environmental impact.

The evolution of the steady DF (see Data availability) and FTIR results (see Figure 3) reveal that the Sol-Gel release agent cannot be easily removed from the plates. 

**Figure 11.**
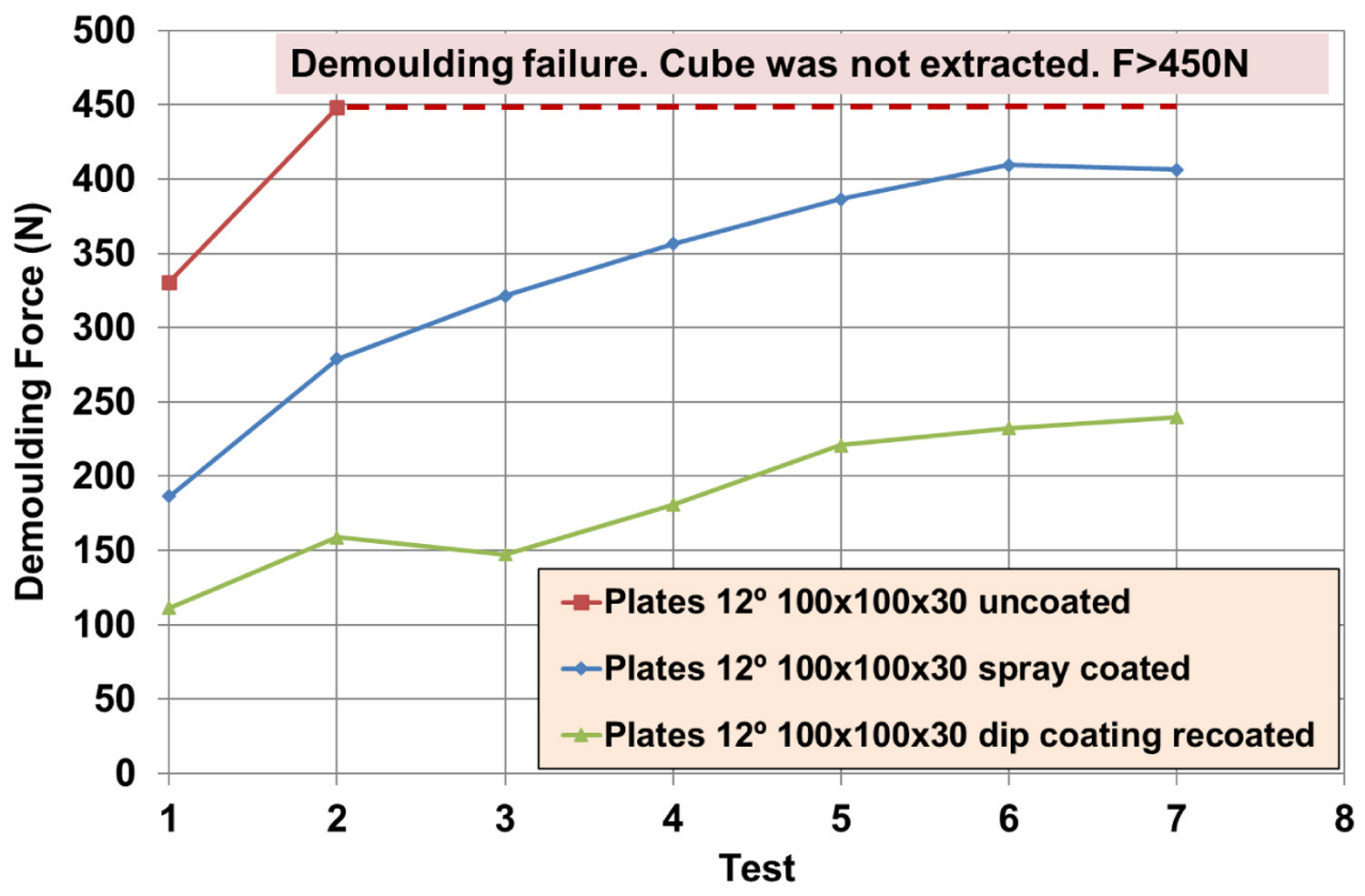
Demoulding force (DF) for FKM90A material: uncoated, spray-coating and dip-coating mould.

**Figure 12.**
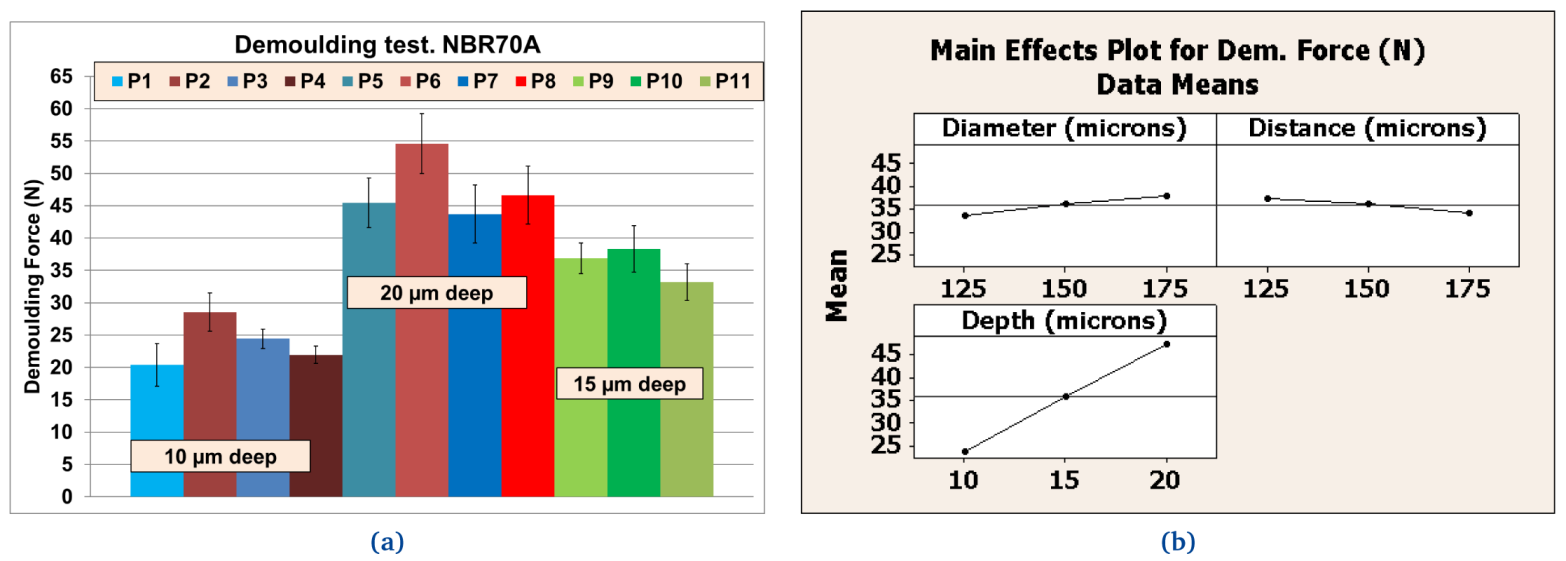
Demoulding forces (DF) for NBR70A material. **a**) Values [N] for different textures; **b**) Effects for texture’s diameter, distance and depth according to references listed in Table 7.

**Figure 13.**
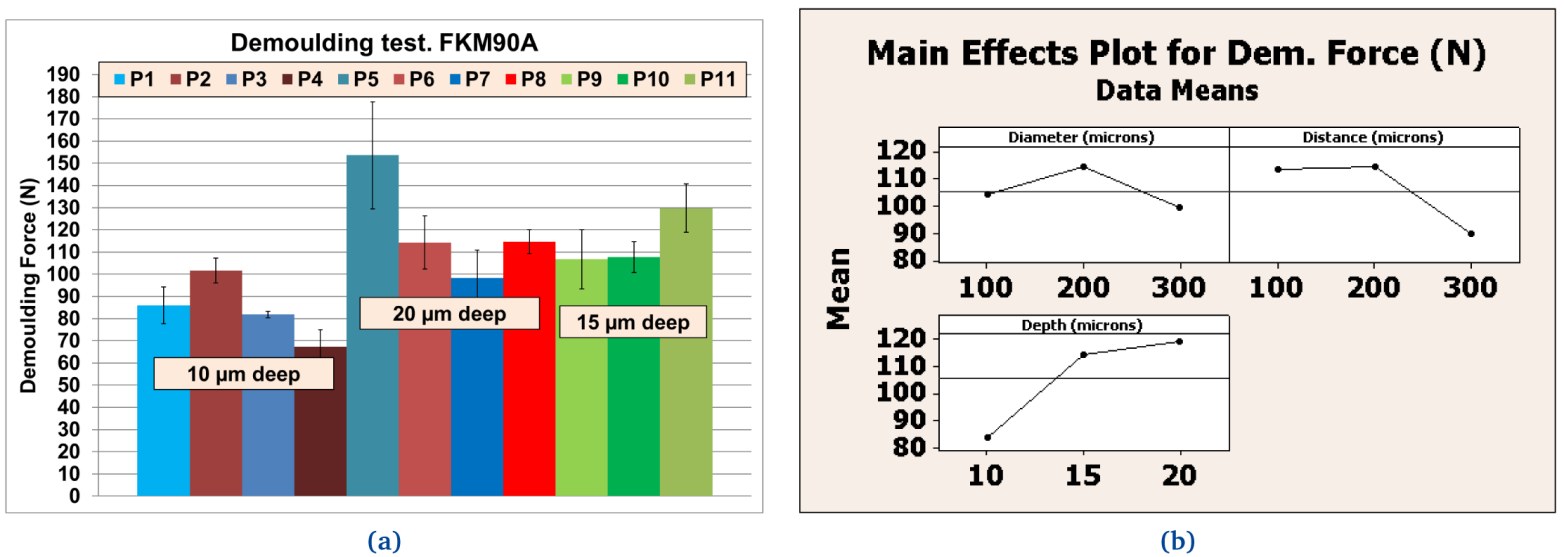
Demoulding forces (DF) for FKM90A material. **a**) Values [N] for different textures; **b**) Effects for texture’s diameter, distance and depth according to references listed in Table 7.

For the FKM90A material, the MT-P coating, a hybrid Sol-Gel formulation with PDMS (no FS) was studied. The influence of the hybrid Sol-Gel application method was analysed on a plate of DDD 100x100x30 µm. In this case, a comparison between uncoated, spray-coated and dip-coated moulds was also performed. The reduction in DF was 70% higher for the dip-coated plates than for the spray-coated ones. Dip-coating method allows the achievement of uniform thickness of the coating layer on the surface (see Figure 3) and better adhesion to the metal substrate. Moreover, it should be remarked that, if any coating is applied, it is not possible to demould the rubber cube after the second test, as revealed by the demoulding failures achieved from second tests for uncoated plates (see red line in Figure 11). The reduction in DF in a textured plate, around 40%, was similar to the results obtained on flat plates for some chemical formulations (see Table 10: Plate 1 and Plate 9). Results in Figure 12 and Figure 13 were limited to 20 µm depth, because experimental evidence for 30 µ depth texture patterns revealed increasing values of DF after seven repetitions (see Figure 11), which gives rise to problems when intended for mass production.

### Texture pattern evaluation

Further tests were performed with different textured plates after coating. The better performing inorganic Sol-Gel (FS-3) was used with NBR70A rubber and the better performing hybrid Sol-Gel (MT-P) was used with FKM90A rubber. It can be observed that the DF for the textured plates were up to three times higher than for the flat ones. For NBR70A material, the DF varied between around 20 N and 60 N, depending on the texture of the coated mould ( Figure 12a), while for FKM90A material, the DF varied between around 65 N and 150 N, depending on the texture of the coated mould (see Figure 13a).

According to the statistical analysis performed on both NBR70A and FKM90A compounds, the DF variation was observed to depend most strongly on the depth of the dimples: higher depth produced greater DF, as expected. The DF were two times higher for NBR70A rubber when minimun and maximun depth were compared. The DF were 1.5 times higher for FKM90A rubber when minimun and maximun depth were compared. DFs showed a linear trend with depth in the case of NBR70A material. Moreover, greater diameter and distance between dimples (lower dimples density) seemed to reduce the DF.

Measured DFs were up to three times higher for FKM90A rubber (more sticky behaviour) than for NBR70A rubber. The observed differences in demoulding behaviour for the two analysed materials can be influenced by elongation properties, where NBR70A rubber shows an almost 5 times higher elongation than FKM90A rubber (see Table 1). Demoulding of textured rubber (positive texture) against textured mould (negative texture) at 0º appeared easier for rubber materials with higher elongation. Taking into account the stress-strain curve of these compounds, this demoulding behaviour is probably due to the lower local stresses around the dimples for a similar local strain in both materials.

### Evaluation of materials with varying hardness

A clear dependence of the DF on both texture and rubber material was observed. In order to analyse the influence of the various rubber compounds (with different hardness) on the DF, one of the plates ( Table 7, Plate 9, DDD 200x200x15µm) was selected to carry out the following tests. Plate 9 was coated with hybrid Sol-Gel MT-P.

**Figure 14.**
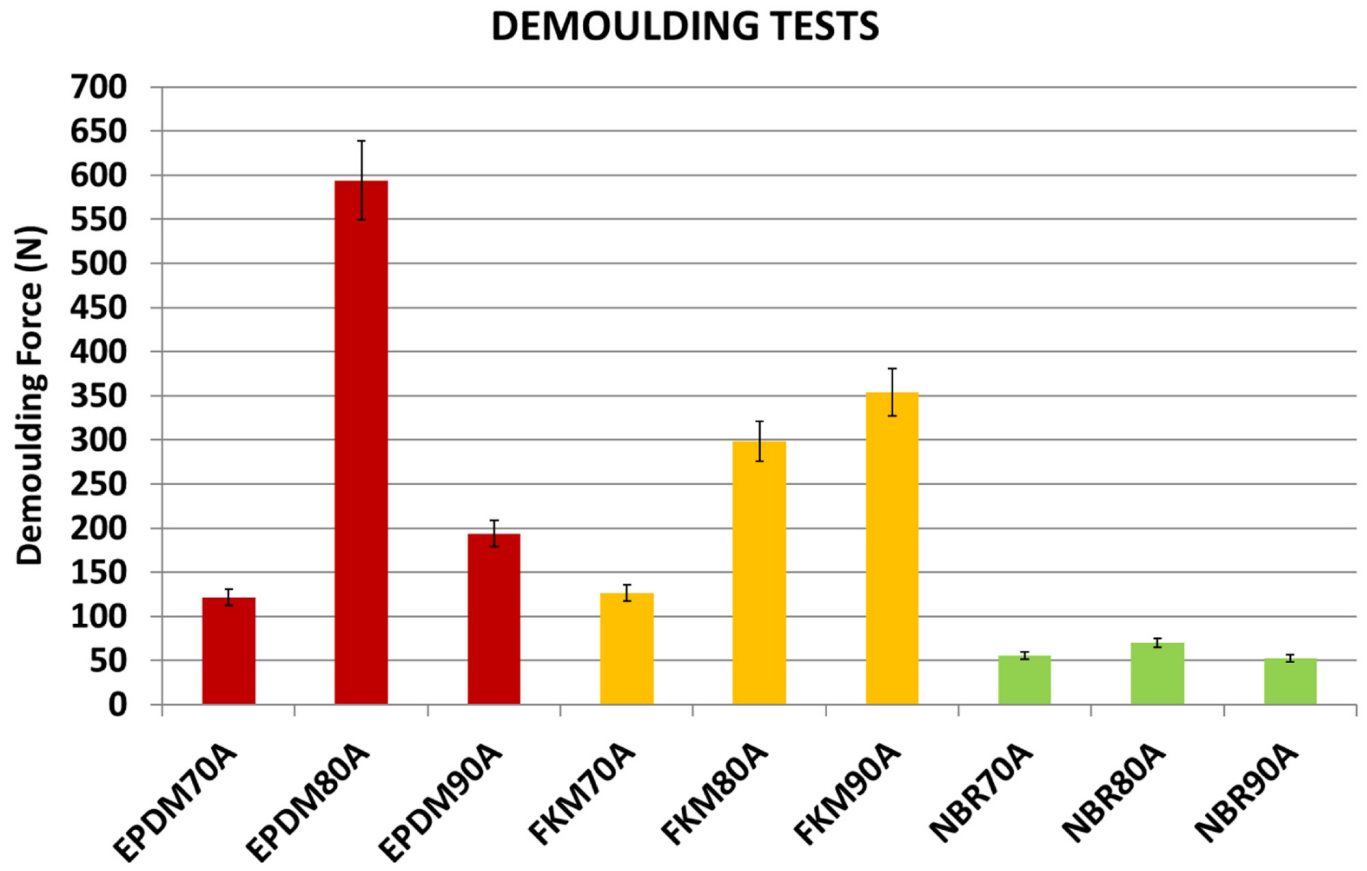
Demoulding forces (DF) for different materials. Diamater, Distance, Depth (DDD) pattern 200x200x15 µm.

**Figure 15.**
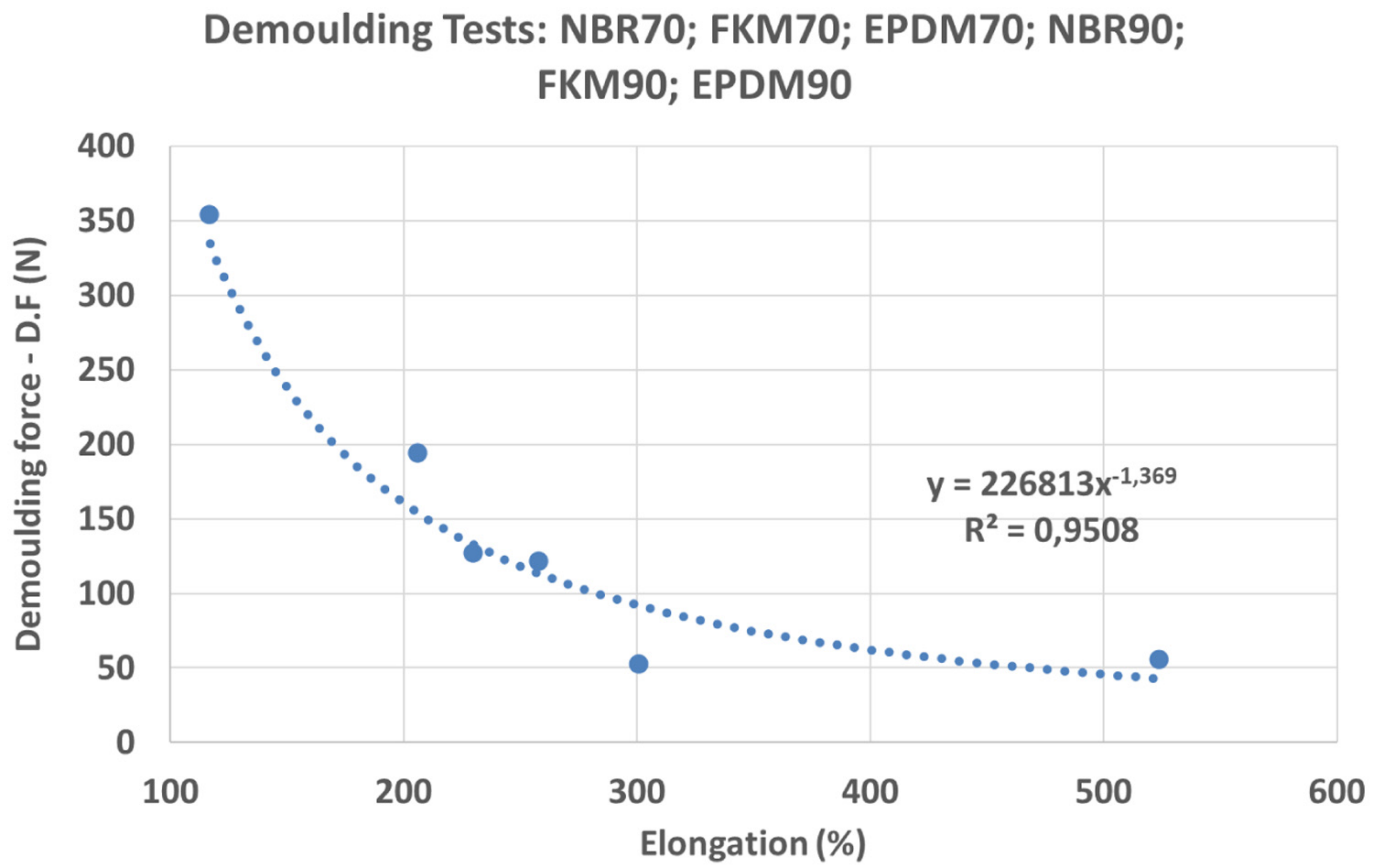
Demoulding Force (DF) vs Elongation.

Results showed remarkable differences between NBR, EPDM and FKM compounds in the DF (see Figure 14). Moreover, an unexpected behaviour was observed with higher DF for EPDM80A: some of the test samples could not be demoulded due to high DFs. For NBR, FKM and EPDM compounds, and shore hardness A 70 and 90, a strong correlation between DF and elongation was observed (see Figure 15): compounds with a higher elongation capability showed lower DFs (i.e. the friction was lower during the demoulding process) and the equation obtained for the potential trend curve was *y* = 226813 *x* ^−1.369^ with *R* ^2^ = 0.95. However, further research needs to be done in order to confirm these results, due to discrepancies with some compounds (shore hardness A 80). From these results, it may be concluded that rubber materials with higher elongation properties (see Table 1) are suitable for a wider range of DDD patterns. Regarding the other investigated variables, no correlation was observed between DF and material hardness nor with rheology parameters. In the case of compounds with Shore hardness A 80, especially for the EPDM80A rubber, the increased DF was linked with a polymer and coating adhesion increment. The analysis of the rheology behaviour of the EPDM80A compound revealed differences compared to the other studied materials (higher ML; higher curing time - t90; lower specific gravity).

After performing more than 40 tests, MT-P coating was still detected in the plates by FTIR analysis. These results are in agreement with those presented in Table 5.

### Roughness influence and manufacturing

**Figure 16.**
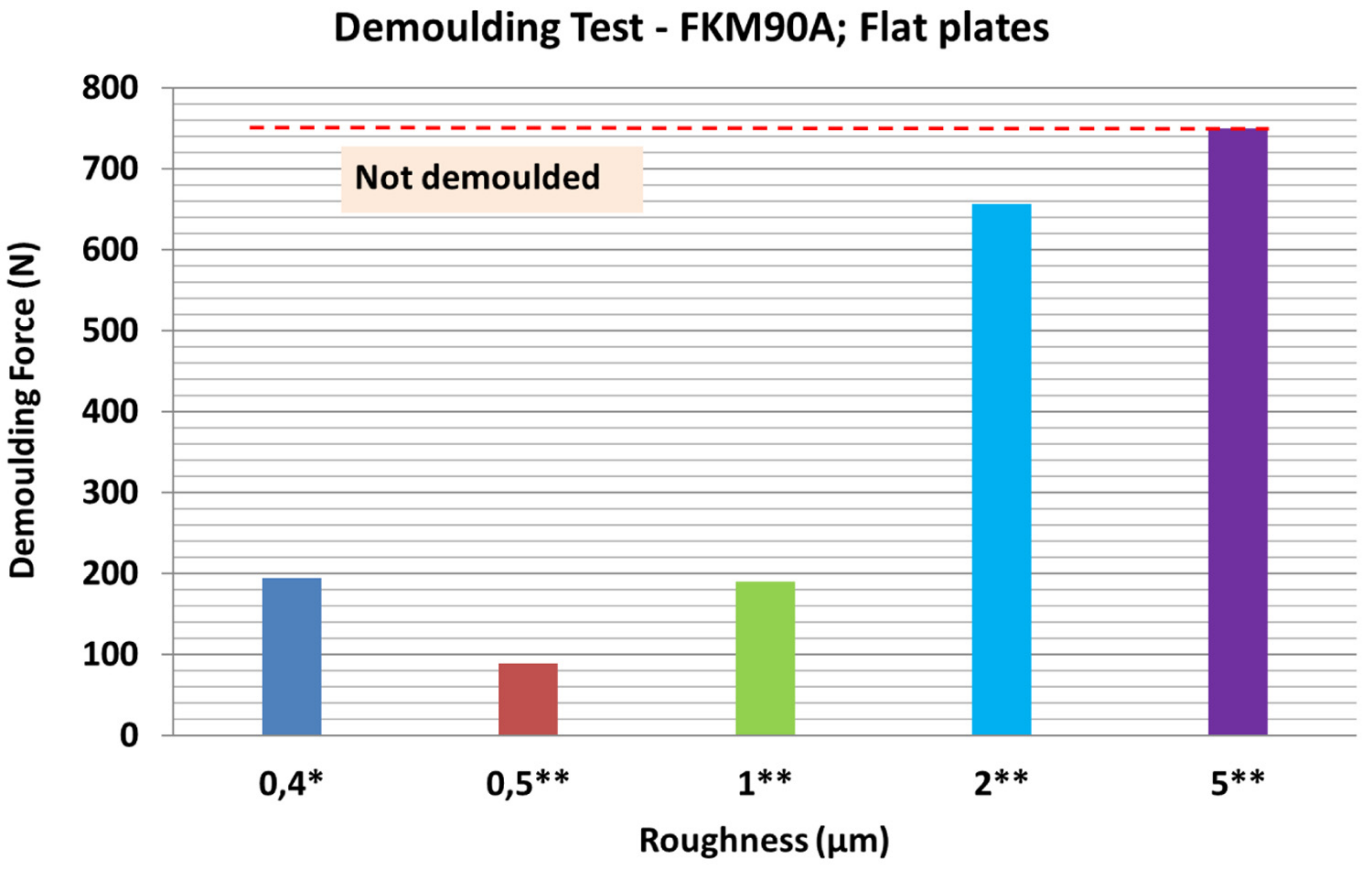
Results of demoulding forces (DF) for different plates roughness values. MT-P Sol-Gel. Milling and drilling machining (*); Laser machining (**). Rubber material with 5 µm roughness could not be demoulded.

Figure 16 shows the results of demoulding tests with a different batch of FKM90A rubber. When the R *_a_* roughness obtained by laser engraving was lower than 1 µm, the DF appeared to remain stable; for plate 2 (0.5 µm roughness) the DF was lower than in the reference case. On the other hand, when laser roughness was above 2 µm, the DF significantly increased; specifically, for 2 µm roughness, it was possible to demould only two rubber cubes and for 5 µm roughness, the sample could not be demoulded.

A linear correlation between the DF and mould surface finishing, in terms of roughness, was observed. According to these results, it is suggested that the increase in the DF was mainly due to friction increment when roughness increased, since no extra adhesions were reported.

Additionally, the findings suggest that laser engraving could be an interesting alternative to machining or sandblasting processes to control roughness on metal plates, and facilitate demoulding mechanisms, which may significantly improve the mould performance. 

### Reduced order modelling results

The eleven plates used in the experiments of Table 7 were grouped according to texture pattern, so that plates 9 to 11 (the three of them with the same dimensions) were considered within the same sample (see Figure 12a and Figure 13a); although previous statistical analyses considered these three samples separately (see Figure 12b and Figure 13b).

**Figure 17.**
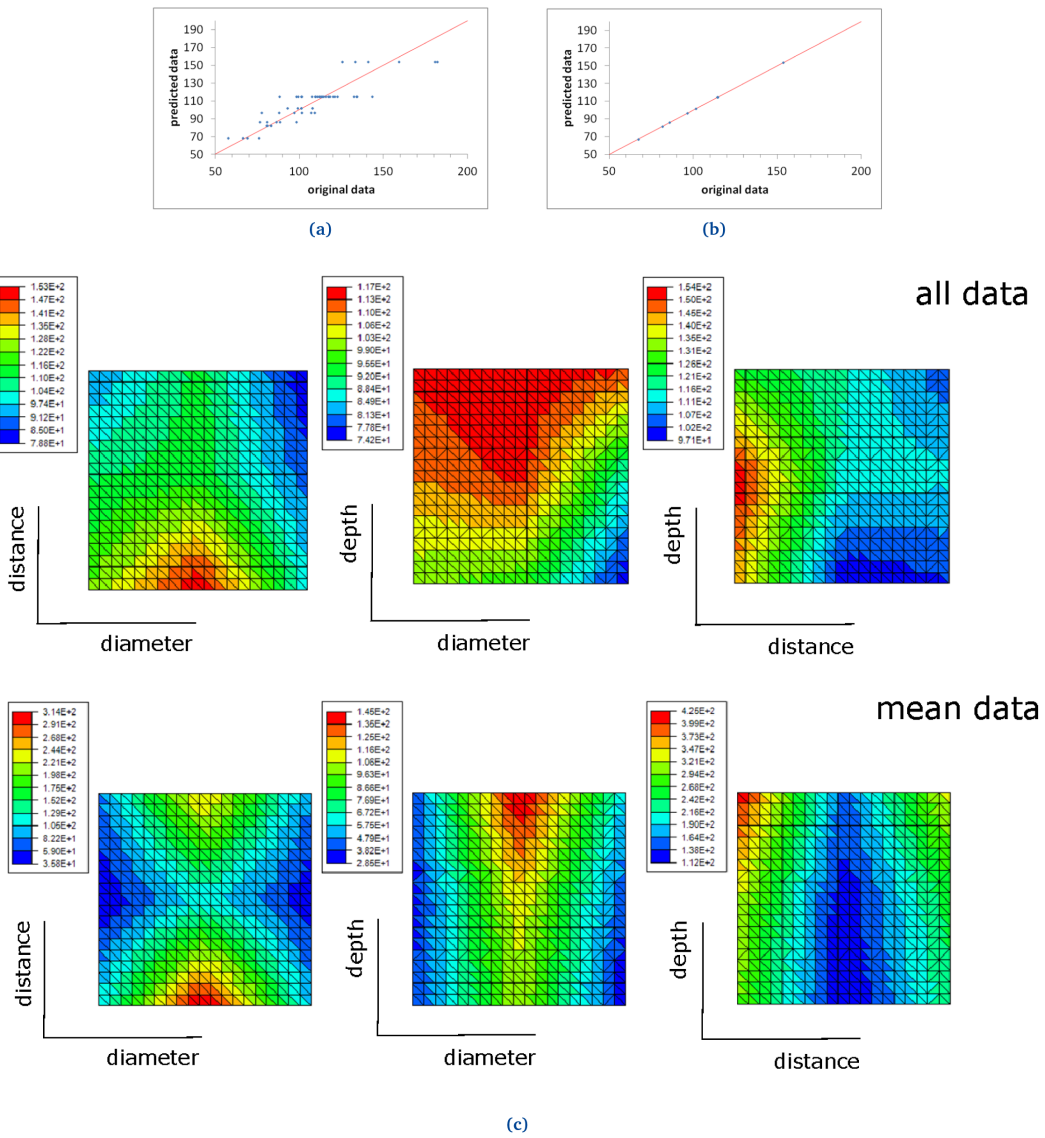
FKM90 ROM prediction with ( **a**) 11 terms, when all demoulding force data are used and ( **b**) 3 terms, when mean demoulding force values are used. The red line in ( **a**) and ( **b**) shows the ideal prediction, i.e. when the predicted data perfectly matches the original ones. FKM90 response surfaces for demoulding force ( **c**). The response surfaces include graphics of "all data" (i.e., no mean values nor standard deviations are considered) and "mean data" (see top and bottom graphs respectively).

**Figure 18.**
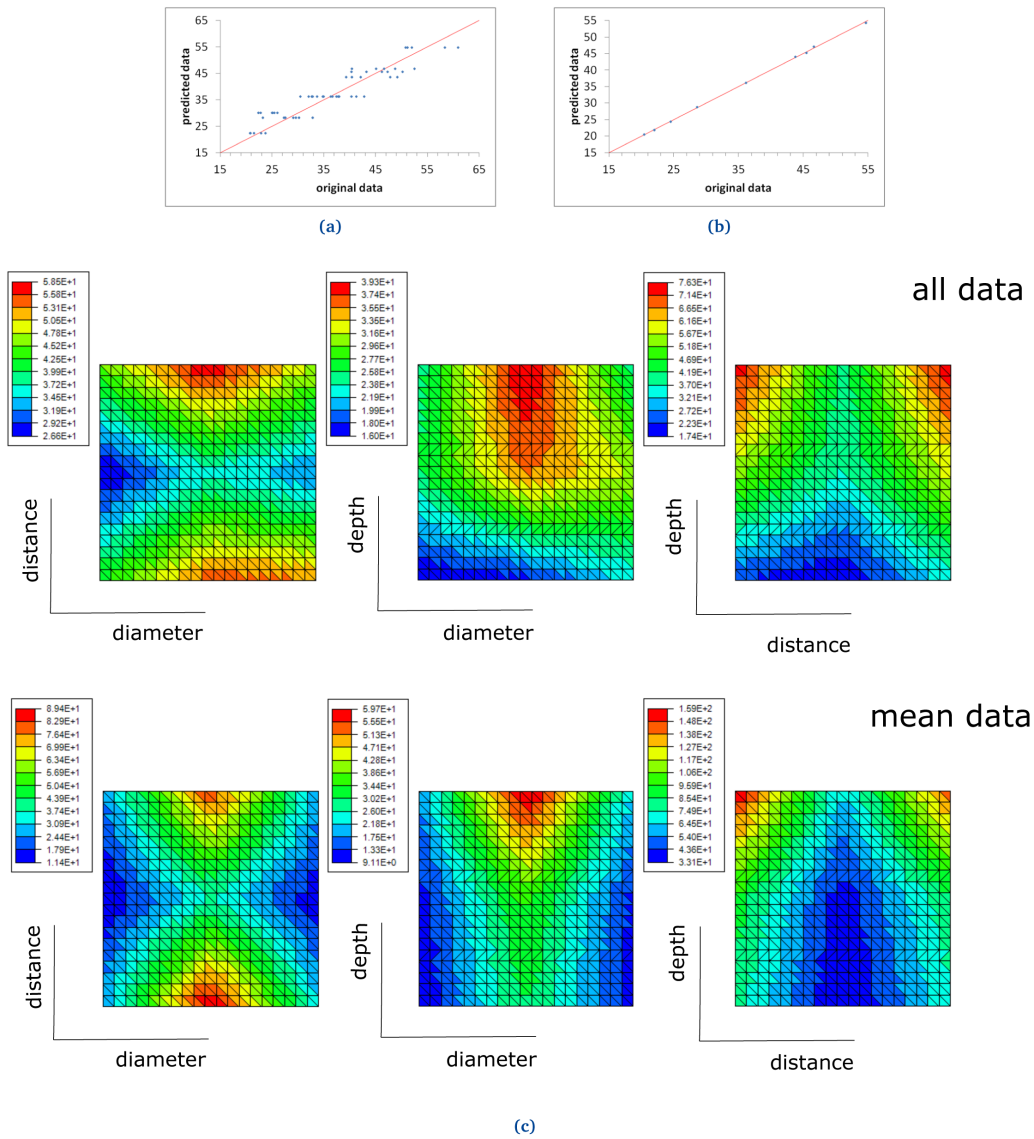
NBR70 ROM prediction with ( **a**) six terms, when all demoulding force data are used and ( **b**) two terms, when mean demoulding force values are used. The red line in ( **a**) and ( **b**) shows the ideal prediction, i.e. when the predicted data perfectly matches experimental data. NBR70 response surfaces for demoulding force ( **c**). The response surfaces include graphics of "all data" (i.e., no mean values nor standard deviations are considered) and "mean data" (see top and bottom graphs respectively).

Two ROM models were computed for two different materials: FKM90A and NBR70A, in order to study the DF, as presented below (see Figure 17 and Figure 18).

### FKM90A and NBR70A ROM results

A first ROM was computed using all DF data, where mean values and standard deviations were not considered ( Figure 17a and Figure 18a). The result converged using 11 terms for FKM90 rubber and six terms for NBR70 rubber, both being the first term over one order of magnitude greater than the next one. The trend line for FKM90 data, shown in Figure 17a perfectly matched the ideal one, i.e. *y* = *x* with *R* ^2^ = 1; although significant scatter was observed. For NBR70 rubber data (see Figure 18a) the equation obtained for the trend line was *y* = 0.93 *x* + 2.27 with *R* ^2^ = 0.87, which shows a satisfying though lower correlation, as well as significant scattering.

A second ROM was computed for the mean values, which converged in only three terms in the case of FKM90 rubber, and two terms for NBR70 rubber; both were once again the first term over one order of magnitude greater than the next terms, in both cases. The predicted data perfectly matched the original experimental results, as shown in Figure 17b and Figure 18b, for both materials respectively. The trend line for FKM90 data, Figure 17b showed a correlation factor *R* ^2^ = 1, where a slope of 1.01 and an intercept of -0.57; for NBR70 rubber data, Figure 18b, the trend line showed *R* ^2^ = 1 as well, with a slope of 1 and intercept of 0.04.

The previously described ROM results were also plotted through response surfaces, where the DF lays on the z axis and two variables were plotted on the x-y plane. Figure 17c shows the response curves obtained when all FKM90 rubber data, i.e. no mean nor standard deviations, and mean demoulding force data are used. Figure 18c shows the same results for NBR70 rubber data. Specifically, Figure 17c and Figure 18c top graphs (all data) show the DF value when two parameters (any two of diameter, distance and depth alternatively) were varied for FKM90 and NBR70 compounds respectively. For FKM90 rubber, it can be concluded that the DF was highest when the dimple distance was relatively small and when the dimple depth increased. No clear trend between the DF and dimple diameter was observed. On the other hand, for NBR70 rubber, extreme distance values together with high dimple depth and mid diameter appeared to increase the DF. Furthermore Figure 17c and Figure 18c bottom graphs (mean data) show similar results as the ones displayed on Figure 17c and Figure 18c top graphs. For both materials, i.e. FKM90 and NBR70, it can be concluded that the DF was maximum when the dimple distance was small and the dimple depth was greater. In particular, the DF increased for greater depths, small distances and medium diameters. In spite of the worse ROM results obtained when raw data were used and no mean DF were considered ( Figure 17a and Figure 18a), the obtained trend results were similar to the ones obtained from the ROM computed with mean DF values, i.e. Figure 17b and Figure 18b. Furthermore, similar conclusions are drawn in comparison to the analysis presented for FKM90 rubber (see Figure 13b).

## Discussion

In this paper, an analysis of the following aspects of the demoulding process is presenteded: the influence of Sol-Gel coatings, dimple-based texture patterns, rubber materials and roughness. The studies were performed through experimental characterizations with a demoulding device operated by a UTM to give insights that facilitate mass production of textured rubber components. Laser engraving of the metallic plates was performed to achieve the desired texture designs. Sol-Gel coatings, used as a release agents, were applied by dip-coating. Negative textures were transferred during moulding to the rubber compound.

The experimental set-up allowed to optimize the chemical formulation of a Sol-Gel coating used as a release agent, where reduction of DF of up to 50% were observed in flat plates. Continuous monitoring of the DF allowed the optimization of the chemical formulation of the Sol-Gel, where fluorinated derivatives were minimized to improve the LCA and reduce costs. Deposition methods of Sol-Gel were analysed and better results were found for the dip-coating application method. Hybrid Sol-Gel with PDMS was selected for industrial applications. Besides, Sol-Gel coatings reduced the DF by up to 40% in textured plates, and the durability of coatings was increased up to 10 times compared to commercial spray release agents. A strong correlation was found between texture depth and the DF, which could represent the main limitation on industrial texturing. Moreover, it was observed that the different elongation values for the studied materials were linked to the DF values: in the case of NBR compounds, the elongation properties ( *>* 250%) allowed a wide texturing range; in the case of FKM compounds, the elongation properties ( *<* 250%) limited the texturing range. One batch with a significantly different behaviour was detected for the EPDM80A compound, which confirms the sensitivity and benefit of using a demoulding test rig to evaluate this process in a laboratory, before starting industrial moulds production and texturing. Finally, the DF was strongly influenced by the roughness of the mould; in particular, for FKM90A rubber, an increase of 1 µm roughness provoked an increment of the DF above 300 N.

The data obtained from the demoulding experiments were useful to compute two different ROM models for the mean values of the DF, for the two studied materials. On the other hand, the intra-geometrical variations observed during the experiments and the reduced amount of data, made the computation of the absolute values of the force challenging, as shown in Figure 17a and Figure 18a.

The demoulding device operated by a UTM was highly useful in terms of scientic information. Furthermore, future modifications of the demoulding device can enlarge their use. Firstly, this study is limited to thermosetting materials with the same temperature of transference chamber and textured mould; however, future modifications (heating by electrical resistance) of the current demoulding test rig will allow to study thermoplastic materials with different temperatures between transference chamber and mould. Secondly, machining of metal plates to avoid burrs, feeding raw material, disassembling and cleaning operations of the demoulding test rig are not easy tasks, which leads to loss of time and temperature conditions (new design of the transference chamber). Thirdly, the geometry of the mould (cube) shows some limitations; curved surfaces, different demoulding angles, simple components (seals) and a comparison between inner and outer texturing should also be studied (new design of the mechanical mould).

All mentioned limitations will be considered in a new demoulding test rig to simplify and widen its use. Future work under the H2020 New Skin project will address several aspects such as increasing the portfolio of rubber materials, moulding real-textured components (such as O-rings, where more accurate information of the demoulding process could be obtained), implementation of an experimental demoulding process with a new demoulding test rig able to manufacture the final geometry of elastomeric seals and measure the demoulding process. The development of a holistic design software has already been started at ITAINNOVA and preliminary results have been obtained. The software, based on ROM results for different rubber materials, geometries, operating conditions, and textures, is proven to be a powerful tool to compute real-time predictions for both friction and DF values ^4, 43^.

## Conclusions

Micro-texturing of rubber components is a complex process when a mass production system is intended; transfer during moulding involves different technologies, like 3D laser micro-texturing of moulds and nano-coatings that must be adequately optimized. In this article, the interaction between textured mould, coating and rubber polymers were investigated to give insights into the different possibilities for industrial texturing in real-world applications. The characterization of demoulding forces in a laboratory with a specific device operated by a universal testing machine gave valuable information that allows a fast and optimized introduction of texturing in rubber components. Finally, the use of reduced order modelling was found to be an useful tool for a fast and precise estimation of the DF on a textured mould for a given geometry and material.

## Data availability

### Underlying data

Zenodo: Demoulding process assessment of elastomers in micro-textured moulds, http://doi.org/10.5281/zenodo.4750927 ^46^

This project contains the following underlying data:

- Material evaluation with different Hardness.xlsx (demoulding forces)
- Roughness influence and manufacturing.xlsx (demoulding forces)
- Sol-Gel evaluation as release agent.xlsx (demoulding forces)
- Texture pattern evaluation.xlsx (demoulding forces)

Data are available under the terms of the Creative Commons Zero "No rights reserved" data waiver (CC0 1.0 Public domain dedication).

## Figures and Tables

**Table:** 

CC	Chemical Composition
CV	Coefficient of Variation
DDD	Diameter, Distance, Depth
DDDR	Diameter, Distance, Depth, Roughness
DF	Demoulding Force
DoE	Design of Experiment
EL	Elongation
EPDM	Ethylene Propylene Diene Monomer
FEA	Finite Element Analysis
FKM	Fluoro Carbon Based Elastomers
FS	Fluorosilane
FTIR	Fourier-Transform Infrared Spectroscopy
HA	Hardness Shore A
Init	Initial peak
LCA	Life Cycle Assesment
LVDT	Linear Variable Displacement Transducer
ML	Moment Lowest
MH	Moment Higest
MPTS	Methacryloxypropyltrimethoxysilane
MTES	Methyltriethoxysilane
NBR	Nitrile Butadiene Rubber
PDMS	Polydimetilsiloxane
R *_a_*	Average Roughness
R *_z_*	Average maximum peak to valley Roughness
Ref.	Reference
ROM	Reduced Order Model
SA	Shore A
SD	Standard Deviation
SG	Specific Gravity
t90	Time to reach 90% of MH
TS	Tensile Strength
TDM	Texturing During Moulding
TEOS	Tetraethoxysilane
TMES	Trimethylethoxysilane
TRD	Tensor Rank Decomposition
UTM	Universal Testing Machine
WCA	Water Contact Angle

## Abbreviations

The following abbreviations are used in this manuscript:
