# Supplementary material for: Demoulding process assessment of elastomers in micro-textured moulds
Source: Open Res Eur. 2022 Feb 24;1:120. Originally published 2021 Oct 6. [Version 2] doi: 10.12688/openreseurope.13716.2 (PMC10446097; doi:10.12688/openreseurope.13716.2)
Supplement: Peer review history

# Peer review: Reviewer response for version 2

**DOI:** 10.21956/openreseurope.15616.r28656

**Published:** 2022-03-17

**Authors:** Nan Zhang

**Affiliations:** 1 Centre of MicroNano Manufacturing Technology (MNMT-Dublin), School of Mechanical and Materials Engineering, University College Dublin, Dublin, Ireland

I am happy with the authors' revision.

Is the study design appropriate and does the work have academic merit?

Yes

Is the work clearly and accurately presented and does it cite the current literature?

Partly

If applicable, is the statistical analysis and its interpretation appropriate?

Yes

Are all the source data underlying the results available to ensure full reproducibility?

Yes

Are the conclusions drawn adequately supported by the results?

Yes

Are sufficient details of methods and analysis provided to allow replication by others?

Yes

Reviewer Expertise:

Micro injection moulding, manufacturing of plastic microfluidic devices, micro/nano manufacturing of surface textures, microfluidic for drug synthesis and diangostics.

I confirm that I have read this submission and believe that I have an appropriate level of expertise to confirm that it is of an acceptable scientific standard.

# Peer review: Reviewer response for version 1

**DOI:** 10.21956/openreseurope.14790.r27778

**Published:** 2021-11-22

**Authors:** Nan Zhang

**Affiliations:** 1 Centre of MicroNano Manufacturing Technology (MNMT-Dublin), School of Mechanical and Materials Engineering, University College Dublin, Dublin, Ireland

The authors have completed a comprehensive analysis of demoulding of rubber materials with micro textured mould. The experimental design is comprehensive and it is well studied. Here are some comments:

1. The authors claimed, “build-up of deposits on the mould surface, after a usually quite small number of moulding cycles, can be another important issue if demoulding forces (DFs) are too high”. But I did not see the relationship between demoulding force and deposition. From my understanding, demoulding force is related only to adhesion and friction. You may need to make it clear on this.
2. The references for micro structures demoulding problem for plastic materials are insufficient.
3. The load cell selected is 100KN, which is much higher than the demoulding forces 10~1000N. Can the authors explain the variation of load during demoulding?
4. It is useful to demonstrate the part morphology with different demoulding forces. It is good to see the relationship between demoulding force and demoulding defects. The author also talked about the increase of demouding forces due to the micro structures. It is important to claim whether the micro structure would be useful in terms of both functional use and manufacturing cost.

Is the study design appropriate and does the work have academic merit?

Yes

Is the work clearly and accurately presented and does it cite the current literature?

Partly

If applicable, is the statistical analysis and its interpretation appropriate?

Yes

Are all the source data underlying the results available to ensure full reproducibility?

Yes

Are the conclusions drawn adequately supported by the results?

Yes

Are sufficient details of methods and analysis provided to allow replication by others?

Yes

Reviewer Expertise:

Micro injection moulding, manufacturing of plastic microfluidic devices, micro/nano manufacturing of surface textures, microfluidic for drug synthesis and diangostics.

I confirm that I have read this submission and believe that I have an appropriate level of expertise to confirm that it is of an acceptable scientific standard, however I have significant reservations, as outlined above.

# Response

**Published:** 2022-01-24

**Authors:** ELIAS LIARTE

**Affiliations:** INSTITUTO TECNOLOGICO DE ARAGON, Spain

First of all we would like to thank you for the comments. They contribute to improve the quality of the paper substantially. Regarding to your questions:

1. The authors claimed, “build-up of deposits on the mould surface, after a usually quite small number of moulding cycles, can be another important issue if demoulding forces (DFs) are too high”. But I did not see the relationship between demoulding force and deposition. From my understanding, demoulding force is related only to adhesion and friction. You may need to make it clear on this.

  Under industrial working conditions, if demoulding forces due to adhesion and friction show high values, build-up of deposits on the mould surface might occur with high probability. Therefore, build-up of deposits on the mould surface can be an important issue for industry, because deposits avoid the correct transference of the geometry. Fortunately, tests with thermosetting materials performed at our laboratory did not show hints for this type of problem, in a limited number of tests.   In order to clarify this, some additional comments have been included in the manuscript.  

1. The references for micro structures demoulding problem for plastic materials are insufficient.

  As the reviewer suggests, more references are included in the manuscript.  

1. The load cell selected is 100KN, which is much higher than the demoulding forces 10~1000N. Can the authors explain the variation of load during demoulding?

  The configuration of the demoulding test rig allows the user to measure all parameters continuously during moulding (compression forces > 20 kN; load cell 100kN) and demoulding (tensile forces < 1kN; load cell 2kN). Since compressive and tensile loads have different order of magnitude, two load cells are used at the same time so as to measure with enough sensitivity. In particular, for the injection of the raw material, forces can be high, up to 50 kN. Otherwise, for the demoulding process, range of forces varies from 10N (NBR70A, flat and coated mould) to 1000 N (FKM90A, nanotextured and uncoated mould) depending on the compound, texture and coating.   Following the reviewer comment, some additional comments on this have been included in the manuscript.  

1. It is useful to demonstrate the part morphology with different demoulding forces. It is good to see the relationship between demoulding force and demoulding defects. The author also talked about the increase of demoulding forces due to the micro structures. It is important to claim whether the micro structure would be useful in terms of both functional use and manufacturing cost.

  I completely agree with your comment and this idea significantly improves the understanding of the paper. Following the reviewer comment, in order to give a first idea about how the morphology of the microstructure can impact the demoulding forces, I have added a new Figure on the manuscript. Additionally, some comments on this have been included in the manuscript. In the new Figure, the influence of the textured pattern in the demoulding force can be observed: Higher density and depth of the texture’s pattern shows higher demoulding forces, although no visual defects were detected in the rubber cubes.

# Peer review: Reviewer response for version 1

**DOI:** 10.21956/openreseurope.14790.r27781

**Published:** 2021-10-25

**Authors:** Frederik Desplentere

**Affiliations:** 1 Research Group ProPoliS, Bruges Campus, KU Leuven, Leuven, Belgium

This paper is focusing on the different techniques to provide a micro structure on a thermoset polymer part within a high throughput process. Different techniques to end up with this microstructure are investigated. For each of these techniques, advantages and disadvantages are indicated. The main focus within this paper is on the investigation of the demolding forces.  The influence of different coating release agents have been investigated and well reported.

 I enjoyed reading this paper. The used materials, methods and processes are well described. The authors also used clear images to explain their research. Only a few minor typos are present. I also indicated some places where a little of extra explanation would help the reader to better understand a specific topic within the paper. This paper is written in a scientific way referring to relevant literature. For me this, paper can be published with minor corrections. I provide the paper with the different remarks in attachment.

Is the study design appropriate and does the work have academic merit?

Yes

Is the work clearly and accurately presented and does it cite the current literature?

Yes

If applicable, is the statistical analysis and its interpretation appropriate?

Yes

Are all the source data underlying the results available to ensure full reproducibility?

Yes

Are the conclusions drawn adequately supported by the results?

Yes

Are sufficient details of methods and analysis provided to allow replication by others?

Yes

Reviewer Expertise:

rheological material characterisation; numerical simulation for polymer processes; experimental validation on industrial equipment using intrumented machines; thermal modelling/processes, polymer engineering from a mechanical point of view, no chemistry backgound

I confirm that I have read this submission and believe that I have an appropriate level of expertise to confirm that it is of an acceptable scientific standard.

# Response

**Published:** 2022-01-24

**Authors:** ELIAS LIARTE

**Affiliations:** INSTITUTO TECNOLOGICO DE ARAGON, Spain

First of all we would like to thank you for the comments. They contribute to improve the quality of the paper substantially. In the updated version, all typos are corrected. Regarding your questions: additives are used to improve hardness and substrate adhesion (titanium, zirconium) and to modify surface energy (fluorine and organosilicons); Figure 4 is updated with a scale; pressure for injection is updated (290 – 800 bar).
